# Co-simulation of human digital twins and wearable inertial sensors to analyse gait event estimation

**DOI:** 10.3389/fbioe.2023.1104000

**Published:** 2023-04-12

**Authors:** Lena Uhlenberg, Adrian Derungs, Oliver Amft

**Affiliations:** ^1^ Hahn-Schickard, Freiburg, Germany; ^2^ Intelligent Embedded Systems Lab, University of Freiburg, Freiburg, Germany; ^3^ F. Hoffmann–La Roche Ltd, pRED, Roche Innovation Center Basel, Basel, Switzerland

**Keywords:** IMU, accelerometer, gyroscope, digital twin, stroke rehabilitation, multiscale modeling

## Abstract

We propose a co-simulation framework comprising biomechanical human body models and wearable inertial sensor models to analyse gait events dynamically, depending on inertial sensor type, sensor positioning, and processing algorithms. A total of 960 inertial sensors were virtually attached to the lower extremities of a validated biomechanical model and shoe model. Walking of hemiparetic patients was simulated using motion capture data (kinematic simulation). Accelerations and angular velocities were synthesised according to the inertial sensor models. A comprehensive error analysis of detected gait events *versus* reference gait events of each simulated sensor position across all segments was performed. For gait event detection, we considered 1-, 2-, and 4-phase gait models. Results of hemiparetic patients showed superior gait event estimation performance for a sensor fusion of angular velocity and acceleration data with lower nMAEs (9%) across all sensor positions compared to error estimation with acceleration data only. Depending on algorithm choice and parameterisation, gait event detection performance increased up to 65%. Our results suggest that user personalisation of IMU placement should be pursued as a first priority for gait phase detection, while sensor position variation may be a secondary adaptation target. When comparing rotatory and translatory error components per body segment, larger interquartile ranges of rotatory errors were observed for all phase models i.e., repositioning the sensor around the body segment axis was more harmful than along the limb axis for gait phase detection. The proposed co-simulation framework is suitable for evaluating different sensor modalities, as well as gait event detection algorithms for different gait phase models. The results of our analysis open a new path for utilising biomechanical human digital twins in wearable system design and performance estimation before physical device prototypes are deployed.

## 1 Introduction

Wearable inertial measurement units (IMUs), among other measuring methods, enable researchers in biomechanics and rehabilitation to measure human kinematics and therefore expand our understanding of natural human movement (Derungs and Amft, 2020; Al Borno et al., 2022). In medicine, movement applications range from analysing walking performance (Balasubramanian et al., 2009), detecting functional impairment in patients after a stroke (Balaban and Tok, 2014), regaining walking autonomy (Canning et al., 2006), to monitoring fall risk in older adults and patients (Tulipani et al., 2020). Yet another application area of IMUs are assistive devices, including the control of orthoses, exoskeletons, and prostheses (Sánchez Manchola et al., 2019). Practical deployment of wearable motion analysis often involves iterative physical testing to optimise and correct sensor positioning (Förster et al., 2009; Niswander et al., 2020), which is tedious due to repeated measurements and prone to error due to, e.g., variance in motion execution, fatigue, loss of motivation, and examiner-dependent errors (Pacini Panebianco et al., 2018; Nilsson et al., 2022). Furthermore, iterative analyses imply, in addition to the considerable time required for study participants/patients and involved experts, that only certain sensor configurations can be evaluated at a time. Simulation-based analyses may offer an excellent tool to systematically address various challenges related to finding suitable sensor configurations and signal interpretation methods from the extensive design space and parametrisation options of wearable IMU systems. Additionally, inter-individual characteristics and coping strategies substantially influence wearable system design and performance of sensor-based movement interpretation (Altini et al., 2015; Derungs and Amft, 2020). For a given wearable application, error effects related to sensor type, body positioning, parametrisation of signal interpretation algorithms, *etc.*, are to date solely available as expert knowledge, if at all. Modeling and simulation methods could enable researchers and practitioners to virtually select, augment, and exchange sensor devices, body positions, as well as to test different modalities and signal interpretation algorithms without patient involvement. Derungs and Amft (2020) performed a first quantitative analysis of gait performance estimation from acceleration signals synthesised in a co-simulation of human body and sensor models. While the work demonstrated that simulation can reveal the full potential of wearable motion sensors and increase their application in clinical practice, the work only focused on dynamic acceleration of the patient’s upper leg and did not cover gait phase events.

**FIGURE 1:**
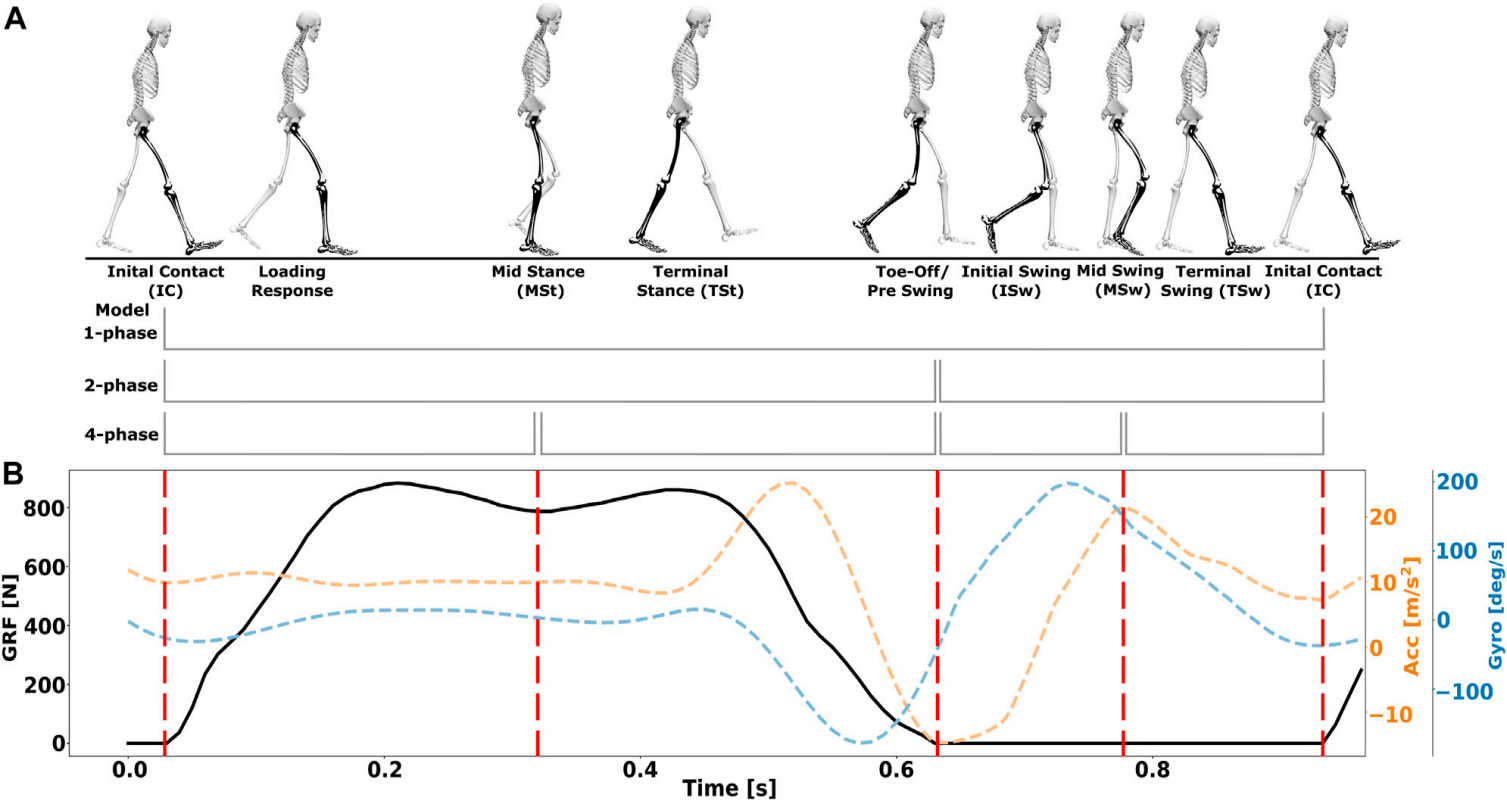
Illustrations of human gait phases. **(A)** Gait cycle division into 1,2,4-phase models. Adapted from (Perry and Bunfield, 2010). **(B)** Visualisation of the four gait events IC, MSt, TO, and MSw aligned with ground reaction force (GRF) data and synthesised accelerometer and gyroscope data of hemiparentic patients from our co-simulation framework.

Gait phase assessment is used clinically to evaluate and diagnose gait disorders, thus helping clinicians to determine and provide optimal care and treatment for patients with walking impairments (Derungs et al., 2018; Han et al., 2019; Sánchez Manchola et al., 2019). Gait phases can be described during a gait cycle from one initial contact to the next initial contact of the same leg (Perry and Bunfield, 2010). One gait cycle consists of two main phases, i.e., stance and swing phase that can be partitioned into eight subphases (Perry and Bunfield, 2010): Initial Contact (IC), Loading Response (LR), Mid Stance (MSt), Terminal Stance (TSt), Pre Swing/Toe off (PSw/TO), Initial Swing (ISw), Mid Swing (MSw), and Terminal Swing (TSw), as shown in Figure 1. A widespread approach to describe gait relies on a 4-phase model (Sánchez Manchola et al., 2019), although events that determine gait phases differ across literature. A variety of published detection algorithms and experimental studies estimated and analysed gait phases, considering various IMU sensors and body positions. Nevertheless, gait detection algorithms were limited to process data derived from a specific body position, preventing performance comparison depending on placement (see also Section 2.3). Furthermore, synthesised sensor data is often used for model training in data augmentation tasks (see also Section 2.1), but the potential of synthesised data for performance analysis of different algorithms, as shown in this work, has hardly been considered. Additionally, there is no methodology to consolidate and analyse performance depending on the choices of algorithms, sensor modalities, and body mounting position, which could improve wearable system design to match applications or individual characteristics.

This paper provides the following contributions:

1. We present a co-simulation framework for synthesising inertial sensor data based on two coupled and time-synchronsed subsystems: 1) personalised inverse kinematic body (i.e., digital twin) and shoe models, and 2) IMU sensor models. Compared to the earlier digital twin approaches, we focus on gait performance analysis of different algorithms and extend our inertial sensor models include triaxial transducers describing static and dynamic acceleration as well as triaxial transducers of angular velocity.
2. We validated our framework against data from a measurement study with healthy volunteers to prove correctness of our modeling and co-simulation approach. Subsequently, we show that the framework is capable of evaluating more challenging gait patterns. Therefore, in simulation experiments, we instantiated a total of 960 IMUs, virtually attached to upper leg, lower leg, and shoe, to analyse gait phase detection of impaired walking in hemiparetic patients.
3. The proposed framework enables system designers to perform a comprehensive error analysis (timing error between detected gait events and reference gait events) of common gait event detection algorithms across 1-, 2-, and 4-phase gait models, considering sensor type and sensor positioning. We varied sensor configuration and algorithm paramerterisation (user-depenent and sensor position-dependent parameterisation) as well as analysed errors that may originate from sensor position variation during wear.

## 2 Related work

### 2.1 Data simulation and synthesis

The concept of human digital twins has already been used, in particular for accurate musculoskeletal models, validated for human motion analysis and simulations. Among others, the open-source platform OpenSim (Delp et al., 2007) has been validated in various human motion studies (Rajagopal et al., 2016; Seth et al., 2018; Karimi et al., 2021; Yap et al., 2021). Biomechanical models provide the basis for evaluating wearable system design, as shown by (Lambrecht et al., 2017; Derungs and Amft, 2020; Mundt et al., 2020; Al Borno et al., 2022). In healthcare and rehabilitation, the digital twin concept is increasingly used, e.g., to partially replace expensive laboratory experiments with *in silico* simulations (Kamel Boulos and Zhang, 2021). Kamel Boulos and Zhang (2021) provided an overview on digital twin concepts used in healthcare. Pizzolato et al. (2019) reviewed neuromusculoskeletal modeling approaches for recovery from spinal cord injury and concluded that the digital twins concept can be applied to people and assistive devices. Derungs and Amft (2020) were the first to leverage digital twins based on personalisable kinematic body models to synthesise sensor data and estimate sensor-dependent algorithm performance in co-simulations.

Collecting patient data can be tedious and error-prone. In addition, many detection models require training data, thus the synthesis of virtual sensor data is gaining interest in the research community. For example, the dataset of Knarr et al. (2013), including walking of eight hemiparetic patients, has been used in previous simulation work by Derungs and Amft (2019). The latter authors demonstrated how biomechanical simulations and synthesised acceleration data could be used to estimate motion-related clinical assessment scores, comparable to data of physical sensors (Wang et al., 2015). Mundt et al. (2020) estimated joint kinematics and kinetics using musculoskeletal modeling to synthesise acceleration and angular velocity data from five selected sensor positions as input for an artificial neural network. Their results showed that the simulation approach is a valid method for data augmentation in biomechanics. Furthermore, generative adversarial networks (GANs), which originally emerged from computer vision research (Zhang and Alshurafa, 2020; Hoelzemann et al., 2021), were proposed to synthesise sensor data for human activities. Zhang and Alshurafa (2020) proposed two deep generative cross-modal architectures for synthesising accelerometer data from video sequences. Kwon et al. (2020, 2021) presented IMUTube, an automated processing pipeline for human activity recognition (HAR) that integrates existing computer vision and signal processing techniques to convert video of human activity into virtual streams of IMU data. A similar approach was chosen by Lämsä et al. (2022), who used neural networks with VIDEO2IMU to generate IMU signals and features from monocular videos of human activities. Their results suggested that HAR systems trained using virtual sensor data could perform considerably better than baseline models, trained using only physical IMU data (Kwon et al., 2021; Lämsä et al., 2022). Esteban et al. (2017) deployed GANs to synthesise respiratory data, where a generator model was used to augment data, and a discriminator attempts to distinguish between real and artificial data. Hoelzemann et al. (2021) used the latter technique with a long-short-term memory layer. Their augmentation approach increased classification performance and produced simulated activity of daily living data similar to data derived from physical measurements. However, there are persisting challenges concerning validity and variability of data augmentation for sensor timeseries, e.g., random transformation is not applicable for every data set, time domain pattern mixing is only recommended for short timeseries or a comparable weak response of timeseries-based neural networks to data augmentation (Iwana and Uchida, 2021). In purely data-driven approaches, e.g., generative models based on neural networks, it is difficult to synthesise new, meaningful time series, as the relation to physical constraints cannot be assessed. In contrast, sensor synthesis from measurement-based inverse kinematic models implements a bottom-up knowledge ordering approach, thus introducing evidence at all model layers. So far, synthesised data was used for model training mostly, but the potential of synthesised data for performance analysis of different algorithms, as shown in this work, has hardly been considered. In this work, we rely on validated biomechanical models that are personalised to synthesise acceleration and angular velocity sensor data on multiple limbs. Compared to previous work, our simulation approach and algorithms aim to evaluate, among a selection of several hundred virtual sensor positions, which sensor configurations and algorithm parameterisations are suitable to estimate gait events.

### 2.2 Sensor positioning

Several approaches have been used to optimise movement monitoring and increase robustness against sensor repositioning. Previous work focused on activity recognition under varying sensor measurements, e.g., considering inter-individual differences and sensor displacement (Lester et al., 2006; Thiemjarus, 2010; Atallah et al., 2011; Banos Legran et al., 2012; Harms et al., 2012; Guo et al., 2016; Chen et al., 2017). Kunze and Lukowicz (2014), described rotation-independent signal features in frequency domain to mitigate sensor positioning effects. Förster et al. (2009) proposed orientation-robust features in addition to self-calibration algorithms to overcome effects of sensor displacement in activity and gesture recognition.

While previous work was limited to physical sensor measurements, Derungs and Amft (2020) investigated sensor positioning and biomarker estimation from synthesised acceleration data, focussing on stride duration, cadence, and step count in athletes and patients after stroke. For hemiparetic patients, differences in gait marker estimation performance were shown, as acceleration sensors were virtually attached to upper legs of affected and less affected body side. Estimation errors ranged up to ∼44% confirmed a sensor position dependency of gait marker estimation. Optimal sensor positions changed over a period of movement therapy. At the less affected body side a favourable ventral region was proposed for sensor positioning. For the affected body side, the area of interest shifted medial-dorsal from before to after an intervention. While the results of Derungs and Amft (2020) showed that personalised and application-adapted wearable systems can considerably outperform non-adapted ones, only dynamic acceleration and selected gait markers were considered. The aforementioned work analysed upper legs of hemiparetic patients only and did not provide a dedicated validation. In contrast, the present work considers complete acceleration and gyroscope sensor models and investigates gait event and stride duration estimation according to basic biomechanical definitions.

### 2.3 Gait event detection algorithms

Due to the clinical relevance and the large number of gait disorders, gait phase analysis with IMUs has already entered practice in research movement labs, e.g., Zhao et al. (2019); Prasanth et al. (2021). Within this context, gait cycle segmentation can be divided into two main categories of applied algorithms (Sánchez Manchola et al., 2019; Vu et al., 2020): rule-based e.g., used by (Catalfamo et al., 2010; Rueterbories et al., 2014; Behboodi et al., 2015; Sánchez Manchola et al., 2019) and data-based, e.g., Hidden Markov Models (HMMs), Artificial Neural Networks and hybrids by (Mannini and Sabatini, 2010; Taborri et al., 2015; Mannini et al., 2016; Zhao et al., 2019), respectively. While HMMs have demonstrated top performance, their training- and time-intensive implementation may be unnecessary (Taborri et al., 2016). Indeed, recent publications show that rule-based algorithms with parameterised thresholds are actively deployed for gait phase detection (Vu et al., 2020; Prasanth et al., 2021). In addition, vertical and antero-posterior linear acceleration and angular velocity at the sagittal plane show periodic and repeating signal patterns over gait cycles, resulting in intuitive, rule-based algorithms (Taborri et al., 2016). Rule-based gait event and phase detection algorithms include time domain-based methods, e.g., (Catalfamo et al., 2010; Rueterbories et al., 2014), time-frequency analysis methods, e.g., (Zhou et al., 2016), and heuristic algorithms, e.g., using derivative peak detection and sign changes (Gouwanda and Gopalai, 2015). Detecting gait events often builds on measurements from gyroscopes and accelerometers individually or in combination (Taborri et al., 2016; Zhou et al., 2016; Vu et al., 2020). For example, Catalfamo et al. (2010) used a rule-based detection approach to segment gait into IC and TO events based on one gyroscope placed at the dominant lower leg. Different inclination levels in seven healthy participants were analysed. The algorithm showed an overall detection accuracy of 99% for level walking. Gouwanda and Gopalai (2015) used a lower leg angular velocity heuristic for real-time detection of two gait phases during walking with and without braces. Although all gait events were detected with an average error of less than 125 ms, abnormal gait patterns were simulated on healthy participants using ankle and knee braces instead of participants with pathological gait. Behboodi et al. (2015) extracted characteristics from lower leg angular velocity signals of four healthy individuals to detect seven gait events with a time difference of approx. 75 ms, and a detection accuracy of 99.8%. Lambrecht et al. (2017) implemented different online peak detection algorithms for segmenting walking in healthy participants. The input signals for each algorithm were chosen from lower leg angular velocity, lower leg segment angle, ankle joint angle, heel linear velocity, toe linear velocity, lower leg position, foot angular acceleration, and toe linear velocity, all obtained in OpenSim. Timing delays ranged between 6 ms and 190 ms, depending on algorithm and gait phase. Sánchez Manchola et al. (2019) proposed a gait phase detection system for a lower limb exoskeleton and evaluated it in nine healthy and nine hemiparetic patients. Vertical acceleration and medio-lateral angular velocity of a single IMU attached to the foot instep was used. They detected four gait phases (IC, FF, HO, TO) with a rule-based algorithm, among others. Compared to reference data from a pressure-sensing insole, the rule-based algorithm showed an overall accuracy of 65.43% and timing errors ranging from −28 ms to 9 ms. Chia Bejarano et al. (2015) placed two IMUs laterally at lower legs to determine angular velocity in the sagittal plane and the lower leg flexo-extension angle. They estimated three gait phases (IC, TO, MSw) with an accuracy of 87% and an average timing difference of up to 52 ms for ten stroke patients with severe impairment. Furthermore, Rueterbories et al. (2014) utilised a rule-based algorithm to identify four gait events from differential acceleration signals of one foot-worn sensor in hemiparetic patients. Their results showed a timing difference ranging from 65 ms to 104 ms, depending on the gait phase.

The above discussion shows that algorithms were restricted to process data derived from a specific body position, thus preventing a comparison of performance depending on placement. Moreover, measurements with several hundred sensor positions simultaneously is prohibitively expensive and not feasible regarding practicality and burden to study participants, e.g., patients. In contrast, our co-simulation framework enables us to investigate motion estimation error using virtually attached sensors at personalised biomechanical models. We demonstrate the co-simulation approach by evaluating estimation algorithms that use vertical acceleration, including static and dynamic acceleration components, as well as medio-lateral angular velocity, across a total of 960 sensors.

## 3 Co-simulation framework

**FIGURE 2:**
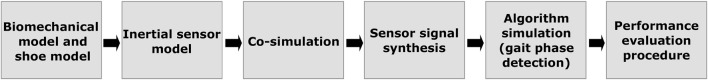
Co-simulation framework overview. Inertial sensor model instances are distributed across biomechanical and shoe models. Motion data is used to co-simulate the models and synthesise inertial sensor data. Based on the various simulated sensors, performance of selected gait event detection algorithms can be analysed in relation to sensor configuration, algorithm parameterisation, and sensor positioning.

We propose a co-simulation framework comprising models to represent the human body as well as inertial sensors. After personalising the body models according to anthropometric data, and defining the inertial sensor placement, models are simulated with motion data (kinematic simulation). Based on the simulation’s kinematic output, inertial sensor signals for each virtual sensor are synthesised. Synthesised data is then fed into selected detection algorithms. Here we focus on gait event detection across 1-phase, 2-phase, and 4-phase gait models. Finally, algorithm performance was analysed in relation to sensor position and gait patterns, to demonstrate the evaluation process based on co-simulation. Figure 2 provides an overview on the co-simulation components and processing steps. In the following subsections, we detail the framework components and their implementation.

### 3.1 Biomechanical body model and shoe model

A biomechanical body model represents the basis for the sensor simulation. We build on personalisable models, e.g., according to anthropometric data, which can be used for motion simulation. In this work, we used the OpenSim (OpenSim, Version 3.3, Simbios, Simbios/SimTK, CA, United States) Gait2354 inverse kinematic model (Delp et al., 2007). Gait2354 is a dynamic 23 degree-of-freedom musculoskeletal human model, including 54 musculotendon actuators of the lower body, torso, and head. The model was validated in healthy participants (John et al., 2013) and different patient groups (Richards and Higginson, 2010; Knarr et al., 2013). Personalisation of the biomechanical body model involved scaling, i.e., changing body mass properties and dimensions, by comparing static distance measurements between specified model landmarks and registering markers (default weights) placed on study participants. Thus, modeling input included body weight and measured motion capture markers.

**FIGURE 3:**
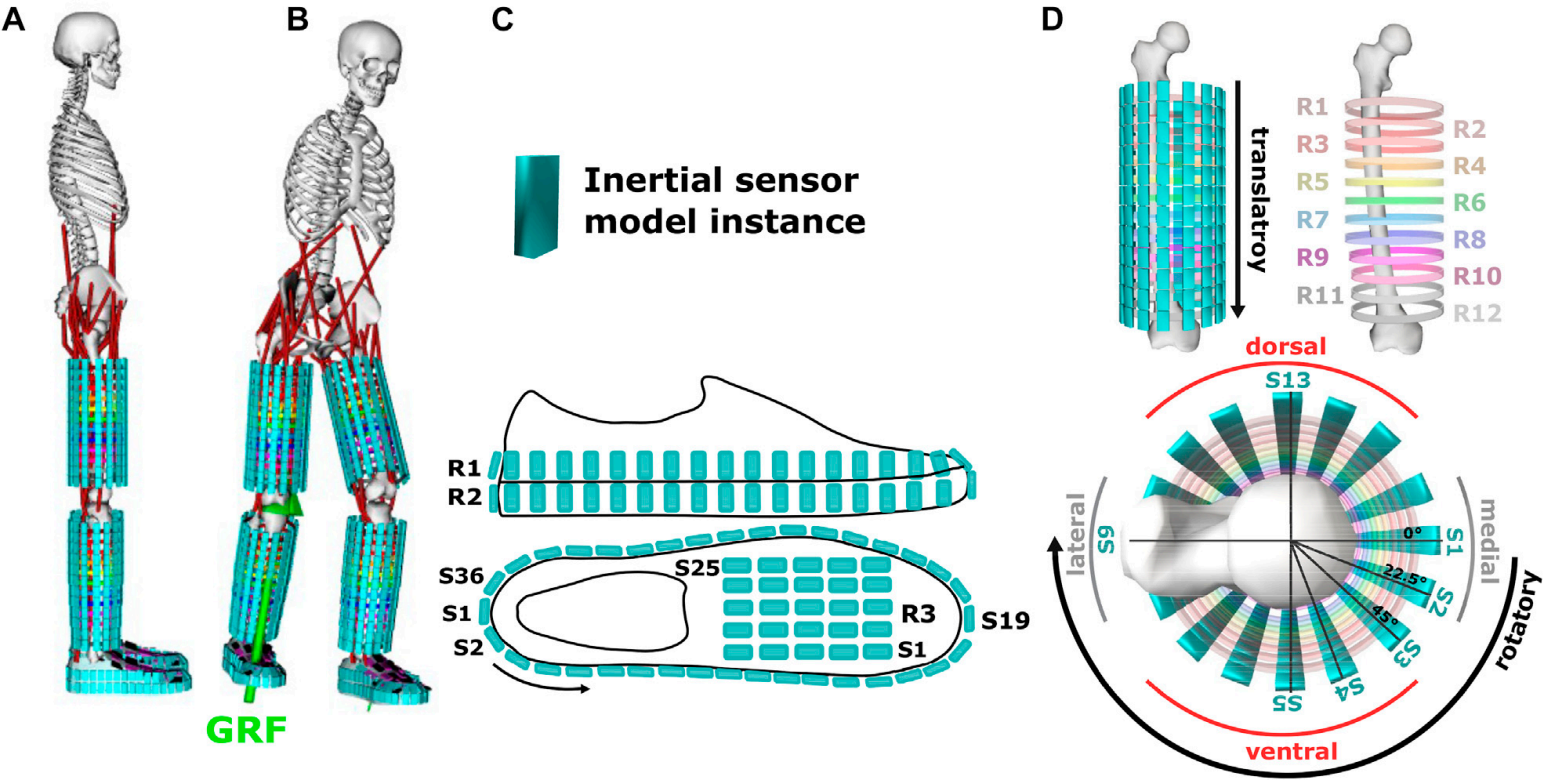
**(A)** Illustration of inertial sensor models attached to a biomechanical body model and a shoe model. The body model uses an auxiliary structure of rings (R) to approximate the limb silhouette. **(B)** Movement simulation inside OpenSim using ground reaction force data (GRF) as reference. **(C)** Shoe sensor positions. **(D)** Sensor positions (S) on the upper and lower legs and visualisation of sensor repositioning along transversal (rotatory part) and longitudinal (translatory part) limb axis of body segments.

Since the original body model represents bones, joints, and muscle links only, an auxiliary structure with a cylindrical shape was designed to approximate limb silhouettes. On the auxiliary structure 12 rings with 16 positions per ring were defined to attach sensors per body side (see Figure 3).

To represent the shoe, an inertia-free, non-deformable, feature-less geometric silhouette model was designed to determine silhouette and sensor positioning (2 rings with 36 sensor positions, and 1 ring with 25 sensor positions). The shoe model was attached to the body model at the foot segment and fixed *via* direct-link (WeldJoint).

A global 3D Cartesian coordinate frame G with coordinate axes (x,y,z) was defined in OpenSim. In addition, Cartesian coordinates of a body-local frame L were defined to support spatial representation of the body and shoe model as anterior-posterior (*x*-axis), vertical (*y*-axis), and medio-lateral (*z*-axis). Initial orientation of the vertical axes of the body-local frame L and the global frame G remained identical throughout all simulations.

### 3.2 Inertial sensor models

An additive model was used to represent 3D acceleration signals, where $\overset{˗}{\;a}\left(t\right)=\left(a_{x},a_{y},a_{z}\right)$ is the sum of the sensor’s dynamic acceleration ${\vec{a}}_{d}\left(t\right)$ and the equivalent gravitational acceleration ${\vec{a}}_{g}\left(t\right)$ acting on the sensor device:

$$\overset{˗}{\;a}\left(t\right)=\;{\overset{˗}{a}}_{d}\left(t\right)+{\overset{˗}{a}}_{g}\left(t\right).$$ (1)

Vector $\vec{k}\left(t\right)=\left(k_{x}\left(t\right),k_{y}\left(t\right),k_{z}\left(t\right)\right)$ is a main surface normal vector at time *t* of an arbitrary simulated sensor instance that is attached at body or shoe models. Vector coordinates were extracted from OpenSim’s ‘Analyse Tool’ using the ‘BodyKinematics’ built-in analysis. Dynamic acceleration $\overset{˗}{\;a_{d}}\left(t\right)$ was synthesised from the second derivative of sensor normal vector $\vec{k}\left(t\right)$ by applying a discrete difference approach.

$$\overset{˗}{\;a_{d}}\left(t\right)=\frac{d^{2}\vec{k}\left(t\right)}{dt^{2}}.$$ (2)

The static acceleration component ${\vec{a}}_{g}\left(t\right)$ was computed by mapping the sensor orientation taken from rotation matrix $Q_{L}^{G}\in R^{3x3}$ to gravity-carrying axis $\vec{j}=\left(0,1,0\right)$ , i.e., along the *y*-axis of the global coordinate frame G. The rotation matrix can be written in matrix form $Q_{L}^{G}\vec{k}$ and rotates sensor position $\vec{k}$ from the global coordinate frame G to the sensor-local coordinate frame L. We assume that the origins of both frames coincide. The resulting unit vector ${\vec{k}}_{j}$ represents the per-axis gravity share:

$${\vec{k}}_{j}\left(t\right)=Q_{L}^{G}\vec{k}\left(t\right)\cdot \vec{j}.$$ (3)

The unit vector ${\vec{k}}_{j}\left(t\right)$ was used to obtain $\overset{˗}{a_{g}}\left(t\right)$ for all sensor axes with *g* = 9.81 m/s^2^:

$${\vec{a}}_{g}\left(t\right)={\vec{k}}_{j}\left(t\right)\cdot g.$$ (4)

Angular velocity, as measured by gyroscope sensors, was synthesised by calculating the first derivative of the orientation estimate with respect to time:

$$\vec{\omega }\left(t\right)=\frac{dQ_{L}^{G}\vec{k}\left(t\right)}{dt},$$ (5)

where $Q_{L}^{G}\vec{k}\left(t\right)$ refers to the simulated sensor orientation, and satisfies ${\left(Q_{L}^{G}\right)}^{-1}={\left(Q_{L}^{G}\right)}^{T}$ and $\det\left(Q_{L}^{G}\right)=1$ according to Zhao (Zhao, 2016).

Sensors were designed as 5 mm^3^ cubes without inertia and scaled in volume by factors of 0.001, 0.005, and 0.003 in x-, y-, and *z*-axis, respectively, to match the scaled biomechanical model in OpenSim (see Figure 3). Our approach was to systematically evaluate position-dependent sensor information. Thus, a systematically arranged sensor grid see Section 3.1 was defined with a maximum distance of 26 mm between individual sensors for a body height of 1.83 m  which corresponds to the tallest participant in our evaluation study. On each upper and lower leg 192 sensors were virtually attached. In addition, 96 sensors were attached to each shoe, resulting in a total of 960 simulated sensors. Sensor attachment at body and shoe models was defined as a direct-link (WeldJoint). Figure 3 illustrates the simulated sensor positions.

### 3.3 Co-simulation and sensor signal synthesis

Co-simulations of the models were performed using OpenSim inverse kinematics tool and motion data as input variables (see Section 5.1). MoCap marker data was filtered by a 6 Hz lowpass Butterworth. Orientation estimates were derived as rotation matrices $\left(Q_{L}^{G}\vec{k}\left(t\right)\right)$ and exported from a customised OpenSim plugin (NIH National Center for Simulation in Rehabilitation Research NCSRR, 2007). From sensor normal vector $\vec{k}\left(t\right)=\left(k_{x},k_{y},k_{z}\right)$ and orientation estimates $\left(\left(Q_{L}^{G}\vec{k}\left(t\right)\right)\right)$ , inertial sensor data were synthesised according to Eqs 1–5. In turn, synthesised sensor data were applied to test the selected algorithms. For algorithm evaluation, we synthesised *y*-axis acceleration $\left({\vec{\;a}}_{y}\left(t\right)\right)$ and z-angular velocity $\left({\vec{\omega }}_{z}\left(t\right)\right)$ , both at 100 Hz.

## 4 Gait detection algorithms

We adapted and contrasted threshold-based gait detection algorithms that were published with promising estimation results using at least one sensor position on the lower limbs. Algorithm (Algo.) A1 used acceleration data only (see Section 4.2.1) and was taken from Catalfamo et al. (2010), but parameterisation, i.e., thresholds were derived from training data (see Section 5.3) instead of visual inspection. We included Algo. A1 as a baseline. Algorithm A2 used gyroscope data corrected by accelerometer data (see Sections 4.2.2–4.2.5). Algo. A2 represents a combination of detection algorithms from Sánchez Manchola et al. (2019) to detect IC and Zhao et al. (2019) to detect Mst and MSw. Similar to Algo. A1, training data was used to derive parameters of Algo. A2. In the aforementioned studies, inertial sensors were attached to feet and lower legs to capture linear acceleration and/or angular velocity signals during gait. Instead we defined multiple sensor positions according to the biomechanical body and shoe models described in Section 3.1.

Synthesised data was preprocessed using a second order Butterworth filter (cut-off: 17 Hz for ${\vec{a}}_{y}\left(t\right)$ and 15 Hz for ${\vec{\omega }}_{z}\left(t\right)$ ) as suggested by (Sánchez Manchola et al., 2019). Data was then filtered with a Savitzy-Golay Filter (fifth order, *w* = 35), to remove outliers, as applied in (Derungs and Amft, 2020). All data analysis was performed using Python 3.8.

### 4.1 Peak detection function

In threshold-based gait event detection, peak detection is a fundamental element. Maxima were detected by a peak detection function $f_{P}^{+}\left(\right)$ in acceleration data ${\hat{a}}_{y}\left(t\right)$ and angular velocity data ${\hat{\omega }}_{z}\left(t\right)$ at each sensor position according to:

$$f_{P}^{+}\left({\hat{a}}_{y},\Theta \right)=\left\{U^{+}\right\}\;and\;f_{P}^{+}\left({\hat{\omega }}_{z},\Theta \right)=\left\{U^{+}\right\}.$$ (6)

Θ refers to parameters of $f_{P}^{+}\left(\right)$ , while *U* ^+^ represents a set of final positive peaks with $U^{+}=\left\{u_{1}^{+},\ldots ,u_{U_{n}}^{+}\right\}$ . Function $f_{P}^{+}$ returns peaks $u_{i}^{+}\in U^{+}$ with timestamp ${\hat{t}}_{i}^{+}$ and signal height ${\hat{h}}_{i}^{+}$ according to:

$$u_{i}^{+}=\left\{t_{i}^{+},h_{i}^{+}\right\}\;for\;i=1,\ldots ,U_{n}.$$ (7)

The peak detection was parameterised by Θ = {*θ*_h_, *θ*_d_} during training, with minimum peak height *θ*_h_ and minimum distance between two consecutive peaks *θ*_d_. Instead of visual inspection to find minimum peak height *θ*_h_, as pursued in previous work (Kotiadis et al., 2010; Maqbool et al., 2016; Sánchez Manchola et al., 2019), in our training procedure, we initially applied peak detection without parameter *θ*_h_, to find all candidate peaks ${\tilde{u}}_{i}^{+}\in {\tilde{U}}^{+}$ . Subsequently, we derived minimum peak height *θ*_h_ as a fraction of the average candidate peak height $\bar{{\tilde{h}}^{+}}$ . For gyroscope signals, we visually chose a fraction of 0.25 and for acceleration signals, a fraction of 0.15, to match the respective signal variability during gait. Corresponding equations are shown in Section 4.2. Average candidate peak height $\bar{{\tilde{h}}^{+}}$ was derived by:

$$\bar{{\tilde{h}}^{+}}=\frac{1}{{\tilde{U}}_{n}}\overset{{\tilde{U}}_{n}}{\underset{i=1}{\sum }}{\tilde{h}}_{i}^{+}.$$ (8)

A wait time *t*_*w*_ between gait events was introduced to avoid bouncing effects and thus false detections, according to (Rueterbories et al., 2013; Sánchez Manchola et al., 2019). Related work described wait times *t*_*w*_, ranging between 40 ms ≤ *t*_*w*_ ≤ 300 ms, e.g., (Maqbool et al., 2016; Sánchez Manchola et al., 2019), or 50% of last stance phase duration (Catalfamo et al., 2010) to detect two to four gait phases. Accordingly, we set *t*_*w*_ = 600 ms for the 1-phase model, *t*_*w*_ = 300 ms for the 2-phase model, and *t*_*w*_ = 150 ms for the 4-phase model.

For minima, peak detection $f_{P}^{-}\left(\right)$ , with $U^{-}=\left\{u_{1}^{-},\ldots ,u_{U_{n}}^{-}\right\}$ was defined accordingly. All peak detections were implemented using detect_peaks from the Python module detecta, which is equivalent to Matlab’s findpeaks() function (Duarte, 2021).

### 4.2 Algorithm-specific parameterisation

Subsequent sections detail specific parameterisation, depending on the algorithm (Algo. A1 or Algo. A2) and phase granularity (1-, 2-, 4-phase see Section 1; Figure 1).

#### 4.2.1 IC event detection used in Algo. A1

Algo. A1 implemented a peak detection to obtain IC from acceleration signal ${\vec{a}}_{y}\left(t\right)$ . Minimum peak height *θ*_h_ of Algo. A1 was set to a fraction of the average candidate peak height $\bar{{\tilde{h}}^{+}}$ in training data following:

$$f_{P}^{+}\left({\hat{\vec{a}}}_{y}\left(t\right),\Theta \right):\;with\;\theta_{h}=\bar{{\tilde{h}}^{+}}-\left(|\bar{{\tilde{h}}^{+}}|\cdot 0.15\right).$$ (9)

Minimal peak distance *θ*_d_ was set equal to *t*_*w*_ = 600 ms between IC events.

The purely acceleration-based Algo. A1 was solely investigated for detection according to the 1-phase model, thus described here for IC event detection only.

#### 4.2.2 IC event detection used in Algo. A2

IC gait events were detected by Algo. A2 by a positive to negative signal sign change function $\left(f_{S}^{Z}\right)$ of the angular velocity signal ${\vec{\omega }}_{z}\left(t\right)$ , followed by a peak in the acceleration signal ${\vec{a}}_{y}\left(t\right)$ with the parameterisation described in Eq. 9. Positive to negative signal sign changes, encoded as ‘1’, were detected according to:

$$f_{S}\left({\hat{\vec{\omega }}}_{z}\left(t_{i}\right)\right)=\left\{\begin{array}{cc} 1,\; & if\;{\hat{\vec{\omega }}}_{z}\left(t_{i-1}\right)>0\;\wedge \;{\hat{\vec{\omega }}}_{z}\left(t_{i}\right)<0\\ 0,\; & otherwise. \end{array}\right.$$ (10)

Algo. A2 reported an IC event, if the sign change and positive peak conditions described above, were met within a time range of 50 ms, according to (Sánchez Manchola et al., 2019). In addition, wait time since the last detected IC event was set to *t*_*w*_ = 600 ms. In the 2- and 4-phase model, we set *t*_*w*_ = 300 ms, *t*_*w*_ = 150 ms, since last detected gait event, respectively.

#### 4.2.3 TO event detection used in Algo. A2

To find TO events with Algo. A2, negative peaks were detected in both, acceleration ${\vec{a}}_{y}\left(t\right)$ (see Eq. 11) and angular velocity signals ${\vec{\omega }}_{z}\left(t\right)$ (see Eq. 12) with a wait time *t*_*w*_ = 300 ms, *t*_*w*_ = 150 ms since the last detected gait event according to 2-, or 4-phase model. We set A2 minimum peak distance *θ*_d_ = 600 *ms* between TO events and minimum peak height *θ*_h_ was set to a fraction of the average negative peak height of candidate peaks $\bar{{\tilde{h}}^{-}}$ in the training data:

$$f_{P}^{-}\left({\hat{\vec{a}}}_{y}\left(t\right),\Theta \right):\;\theta_{h}=\bar{{\tilde{h}}^{-}}+\left(|\bar{{\tilde{h}}^{-}}|\cdot 0.15\right);$$ (11)

$$f_{P}^{-}\left({\hat{\vec{\omega }}}_{z}\left(t\right):\Theta \right),\;\theta_{h}=\bar{{\tilde{h}}^{-}}+\left(|\bar{{\tilde{h}}^{-}}|\cdot 0.25\right).$$ (12)

A2 reported a TO event if ${\hat{u}}_{i}^{a}$ of the acceleration was followed by a ${\hat{u}}_{i}^{\omega }$ of the angular velocity signal within a time range of 50 ms.

#### 4.2.4 MSw event detection used in Algo. A2

MSw was determined from peaks of the angular velocity signal ${\vec{\omega }}_{z}\left(t\right)$ with a wait time *t*_*w*_ = 150 ms since the last detected IC event. Minimum peak distance was set to *θ*_d_ = 600 ms between MSw events. Minimum peak height *θ*_h_ was set as a fraction of the average peak height of candidate peaks ${\tilde{h}}^{+}$ in training data:

$$f_{P}^{+}\left({\hat{\vec{\omega }}}_{z}\left(t\right),\Theta \right):\;\theta_{h}=\bar{{\tilde{h}}^{+}}-\left(|\bar{{\tilde{h}}^{+}}|\cdot 0.25\right),$$ (13)

while the acceleration signal of the contralateral side ${\vec{a}}_{y}^{⋆}\left(t\right)$ exhibited minimal signal changes, determined as gradient (using second order derivative) with a tolerance set at the 75th quantile in signal segments with *t*_*d*_ = 50 ms (Sánchez Manchola et al., 2019). To determine the signal change described above, ${\vec{a}}_{y}^{⋆}\left(t\right)$ was segmented into time windows of *t*_*d*_ = 50 ms (Sánchez Manchola et al., 2019).

#### 4.2.5 MSt event detection used in Algo. A2

MSt was detected by peaks of the angular velocity signal at the contralateral side ${\vec{\omega }}_{z}^{*}\left(t\right)$ with a wait time *t*_*w*_ = 150 ms since the last detected TO event. Minimum peak height *θ*_h_ was set as a fraction of the average peak height in candidate peaks $\bar{{\tilde{h}}^{+}}$ in the training data according to:

$$f_{P}^{*}\left({\hat{\vec{\omega }}}_{z}^{*}\left(t\right):\Theta \right),\;\theta_{h}=\bar{{\tilde{h}}^{*}}-\left(|\bar{{\tilde{h}}^{*}}|\cdot 0.25\right).$$ (14)

We set minimum peak distance *θ*_d_ = 600 ms between MSt events. A2 peak detection reported a MSt event, if the acceleration signal exhibited minimal signal changes, determined as gradient (second order derivative within a tolerance of the 75th quantile) in signal segments with *t*_*d*_ = 50 ms.

## 5 Evaluation procedure

### 5.1 Evaluation dataset

Simulation data was extracted from a publicly available dataset (Knarr et al., 2013), comprising treadmill walking of eight hemiparetic patients after stroke. Patients walked at self-selected speed, before (PRE) and after (POST) an intervention therapy. Biomechanical motion references from marker-based video motion capture and measured ground reaction force (GRF) were used in our work. Our evaluation covers both body sides of hemiparetic patients, i.e., the affected and less-affected body side.

### 5.2 Ground truth

To obtain ground truth, we used GRF data, which provides details about gait phases (see Figure 1). GRF data served as reference to identify IC, MSt, TO, and MSw events for each study participant. IC and TO were extracted automatically by detecting signal sign changes of the GRF first derivative. MSt and MSw were visually labelled by an experienced sports scientist.

### 5.3 Algorithm validation approach

We apply five-fold cross-validation (CV) and Leave-One-Participant-Out CV as validation strategies to assess detection algorithm performance. The validation strategies target different evaluations, i.e., to estimate errors of user-dependent vs. user-independent parameterisation and errors of sensor position-dependent vs. sensor position-independent parameterisation at limb segments (see details below). During training, the respective algorithm parameters to find gait events (IC, TO, MSt, MSw, see Sections 4.2.1–4.2.4) were fitted, while the CV testing sets were used to evaluate performance. All algorithms were evaluated for PRE and POST intervention separately (see Section 5.1). Below we match validation strategies to algorithms, i.e., how minimum peak height *θ*_h_ was derived from training data and evaluated on testing data.

#### 5.3.1 Algo. A1 and Algo. A2a

To assess event estimation performance in user-dependent and sensor position-dependent parameterisation, Algo. A1 and Algo. A2, variant a (A2a), were evaluated using 5-fold CV per participant and sensor position, thus each fold was split into 80% training data and 20% testing data. Training data was used to fit minimum peak height *θ*_h_ according to synthesised sensor data. Training procedure was repeated five times to cover each data fold once for testing and folds’ results were averaged.

#### 5.3.2 Algo. A2b

To assess event estimation performance in user-independent parameterisation, Algo. A2, variant b (A2b), was evaluated using Leave-One-Participant-Out procedure. Training data was used to fit minimum peak height *θ*_h_ according to synthesised sensor data per sensor position, but averaged across all study participants, except one participant. Data of the held-out participant was assigned for testing. Training procedure was repeated to hold-out every participant once and the folds’ results were averaged.

#### 5.3.3 Algo. A2c

To assess event estimation performance in user-dependent, but sensor position-independent parameterisation, Algo. A2, variant c (A2c), was evaluated using 5-fold CV per participant, thus each fold was split into 80% training data and 20% testing data. Training data was used to fit minimum peak height *θ*_h_ according to synthesised sensor data and subsequently averaged per body segment. According to the number of synthesised sensors per segment, 192 sensors at each upper and lower legs, and 96 sensors at the shoe were averaged before applying CV. Training procedure was repeated five times to cover each data fold once for testing and the folds’ results were averaged.

### 5.4 Gait event evaluation

Performance of the gait event detection algorithms was evaluated by deriving timing error, i.e., mean absolute error (MAE) of detected events ${\hat{t}}_{i}$ and reference events *t*_*i*_ (see Eq. 7) across all *U*_*n*_ events per test set following:

$$MAE=\frac{1}{U_{n}}\overset{U_{n}}{\underset{i=1}{\sum }}\left|t_{i}-{\hat{t}}_{i}\right|.$$ (15)

Subsequently, normalised MAE (nMAE) was obtained in percent by normalisation with the reference average stride duration $\bar{F}$ per body side, intervention condition, and patient (see Eq. 16). Reference average stride duration $\bar{F}$ was determined according to GRF reference signal (see Section 5.2).

$$nMAE=\frac{MAE}{\bar{F}}\cdot \;100\%.$$ (16)

To investigate detection errors that may originate from position variation during sensor wearing, we analysed rotatory and translatory error subcomponents for Algo. A2c. Algo. A2c was designed as sensor-position independent, i.e., the same parameterisation for minimum peak height *θ*_h_was used for each sensor position per body segment (see Section 5.3.3). Rotatory errors were determined from estimated errors (nMAE) at sensor positions S1-S16 of auxiliary structure ring R1, see Figure 3D, thus simulating an uniaxial sensor rotation around a limb segment. Correspondingly, translatory errors were determined from estimated errors (nMAE) at sensor position S1 of auxiliary structure ring R1-R12, thus simulating an uniaxial sensor translation along a limb axis. The error components can be interpreted as voluntary or involuntary sensor position shifts on longitudinal and transversal limb axes. By dissecting error components, we assessed how susceptible the detection algorithms are to specific types of sensor position shifts.

## 6 Model validation

The model validation was performed with specifically recorded motion data to confirm correct synthesis of acceleration and gyroscope signals as most public datasets lacked synchronous MoCap marker data and physical IMU measurements, which are needed for a direct comparison. Synthesised and measured (reference) sensor data timeseries of ten healthy participants during walking were analysed to validate our modeling and co-simulation approach.

A total of 24 reflective spherical MoCap markers and six IMUs (MyoMotion, Noraxon, United States) at lower limbs and pelvis were selected. A synchronized and calibrated 11-camera marker-based MoCap system (Qualisys, Sweden) was used to acquire gold-standard MoCap data. Cameras and IMUs were time-synchronized at a frame rate of 100 Hz. See Supplementary Table S1 for participant details and Supplementary Figure S13 for participant setup during model validation. All participants gave written consent and ethics approval was granted by the ethics committee of Friedrich-Alexander University Erlangen-Nuremberg.

After a static calibration trial (upright standing), participants were instructed to walk straight for a distance of 9 m. On average two gait cycles per body side and participant were captured for the analysis. MoCap data preprocessing included marker labelling and gap filling (Qualysis Track Manager, v. 2018). MoCap and IMU data were subsequently filtered by a 6 Hz lowpass Butterworth.

The Gait2354 inverse kinematic model was used to create personalised body models following the steps described in Sections 3.1, 3.3. The best matching sensor positions were selected (determined manually as lowest error and highest correlation across all participants) at the upper limbs (S4 on R12), lower limbs (S3 on R12) and foot (S22 on R3) for comparison with real measurements (see Figure 3).

**FIGURE 4:**
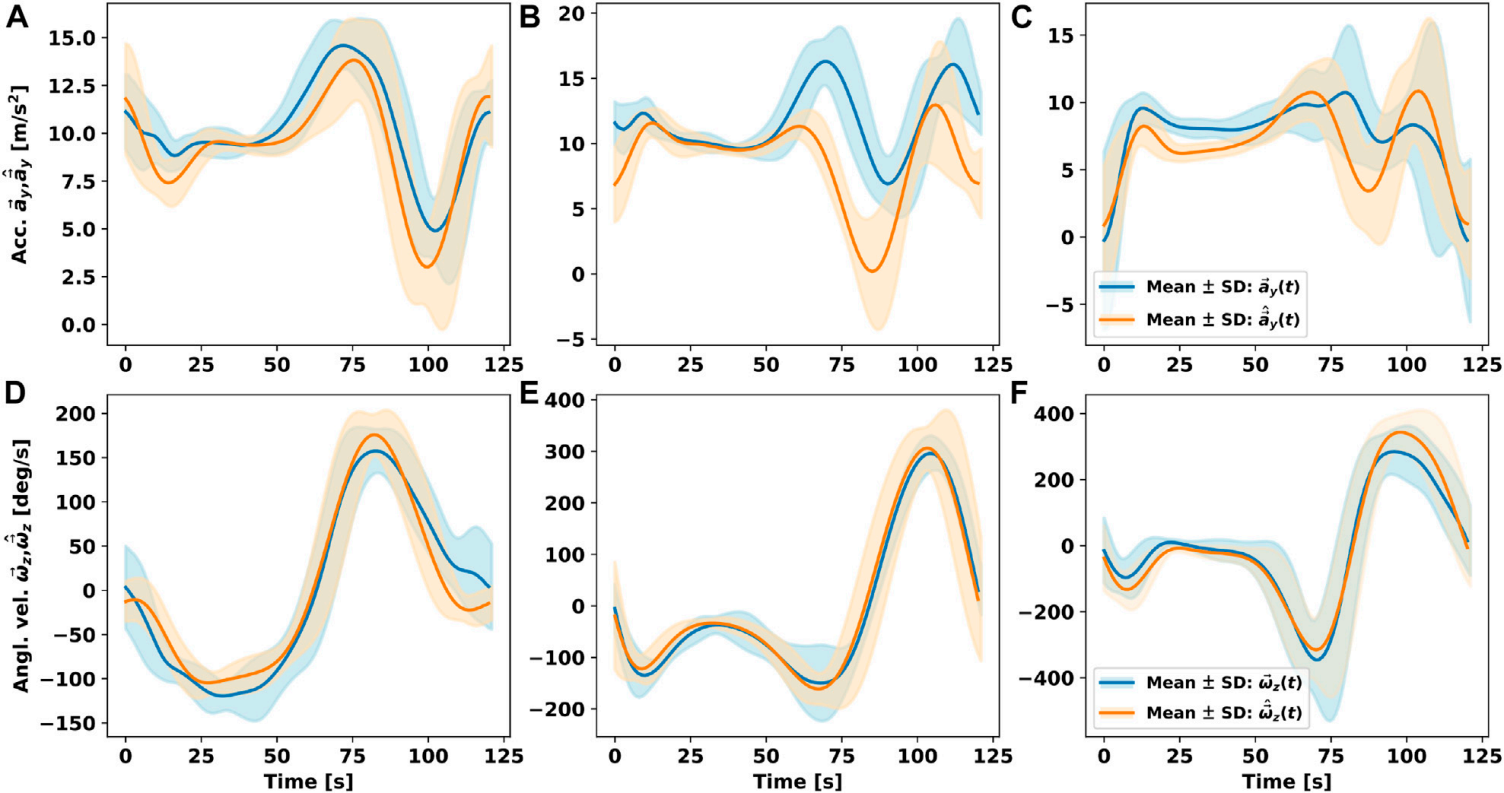
Comparison of accelerometer (Acc.) and gyroscope (Angl. vel.) measurements timeseries $\left({\vec{a}}_{y},{\vec{\omega }}_{z}\right)$ and synthesis timeseries $\left({\hat{\vec{a}}}_{y},{\hat{\vec{\omega }}}_{z}\right)$ . **(A)** Upper leg acceleration. **(B)** Lower leg acceleration. **(C)** Foot instep acceleration. **(D)** Upper leg angular velocity. **(E)** Lower leg angular velocity. **(F)** Foot instep angular velocity.

Figure 4 shows measured and simulated signals averaged per gait cycle for upper and lower legs and foot instep. Data of left and right body sides were combined, resulting in a total of 48 gait cycles per segment to be analysed. Synthesised and measured accelerometer data showed good average agreement at a Pearson’s correlation coefficient of r = 0.62 across all three sensor positions (*cf.* Figures 4A–C). Pearson’s correlation coefficients were derived between synthesised and measured sensor timeseries data per participant and sensor position. All Pearson correlation r values were transformed with the Fisher’s z transform as not all correlations were normally distributed. We calculated mean and 95% confidence interval (lower and upper bounds) from the transformed distributions. Subsequently, the derived means and bounds were inversely transformed (see Table 1). Correlation was *r* = 0.88 for upper legs, *r* = 0.44 for lower legs, and *r* = 0.56 for feet. Absolute signal minima and maxima varied between synthesised and measured timeseries, however, mean median absolute deviation (mean MAD) was 1.51 m/s^2^, mean RMSE was 3.59 m/s^2^, and mean normalised median absolute deviation (mean nMAD) was below 10% (see Table 1). Reduced correlation at lower legs and feet compared to upper legs may be due to the auxiliary structures used to approximate limb shapes: The cylindrical auxiliary structures had larger circumferential difference at lower legs, thus making it harder to map sensor positions, compared to upper legs. Similarly, the shoe model may have resulted in larger variation of foot sensor approximation compared to barefoot walking (*cf.* Figures 4A–C; Table 1). For angular velocity signals correlations were *r* = 0.98 for lower legs, *r* = 0.94 for feet, and *r* = 0.92 for upper legs. Figures 4D–F illustrates the agreement of simulation and measurements. Lowest mean nMAD were 7% for lower legs, overall mean nMAD was ∼10.7% (see Table 1).

**TABLE 1:** Pearson correlation coefficients r, mean MAD, mean RMSE, mean nMAD, and STD between measured and synthesised sensor timeseries across all participants. LO/HI Bound: Lower and upper bound of 95% confidence interval.

		Acceleration				Angular velocity			
		**Upper leg**	**Lower leg**	**Foot**	**Mean**	**Upper leg**	**Lower leg**	**Foot**	**Mean**
**r**	**Mean**	0.88	0.44	0.56	**0.63**	0.92	0.98	0.94	**0.95**
	**LO/HI Bound**	0.83/0.92	0.28/0.58	0.42/0.68	**0.51/0.73**	0.88/0.94	0.97/0.98	0.92/0.95	**0.92/0.96**
**MAD** [ *m*/*s* ^2^; °/*s*]	**Mean**	1.09	1.6	1.85	**1.51**	20.01	15.06	28.44	**21.17**
	**STD**	0.09	0.14	0.08	**0.10**	2.74	3.46	3.63	**3.28**
**RMSE** [ *m*/*s* ^2^; °/*s*]	**Mean**	1.95	5.16	3.66	**3.59**	31.48	25.91	53.52	**36.97**
	**STD**	0.08	0.12	0.26	**0.15**	3.29	3.21	4.71	**3.74**
**nMAD [%]**	**Mean**	5.22	7.2	16.2	**9.54**	9.45	6.18	16.32	**10.65**
	**STD**	0.65	1.49	1.54	**1.23**	3.35	3.69	5.18	**4.07**

Highlight Mean column and Metric used.

Overall, angular velocity signal agreement exceeded that of acceleration. Nevertheless, signal patterns between synthesised and measured acceleration signals clearly showed similarity, too. The results indicate that acceleration is more susceptible to individual movement patterns and potential sensor position differences. An overall agreement between signal patterns is evident, suggesting that our co-simulation framework provides meaningful fidelity for sensor data synthesis of acceleration and angular velocity data.

## 7 Results

We summarise estimation results of gait event detection based on 1-, 2-, and 4-phase models. Result diagrams use a common boxplot style to show error ranges over all simulated sensors per limb, with whiskers extending to 1.5× the interquartile range (IQR) below/above the lower/upper quartile (Q1/Q3). Since error estimates were not always normally distributed, we compare median and IQR, but visualise error means too. We limit visible error ranges to highlight the performance of practically relevant sensors per limb with nMAE below 50%.

### 7.1 Event detection with the 1-phase model

**FIGURE 5:**
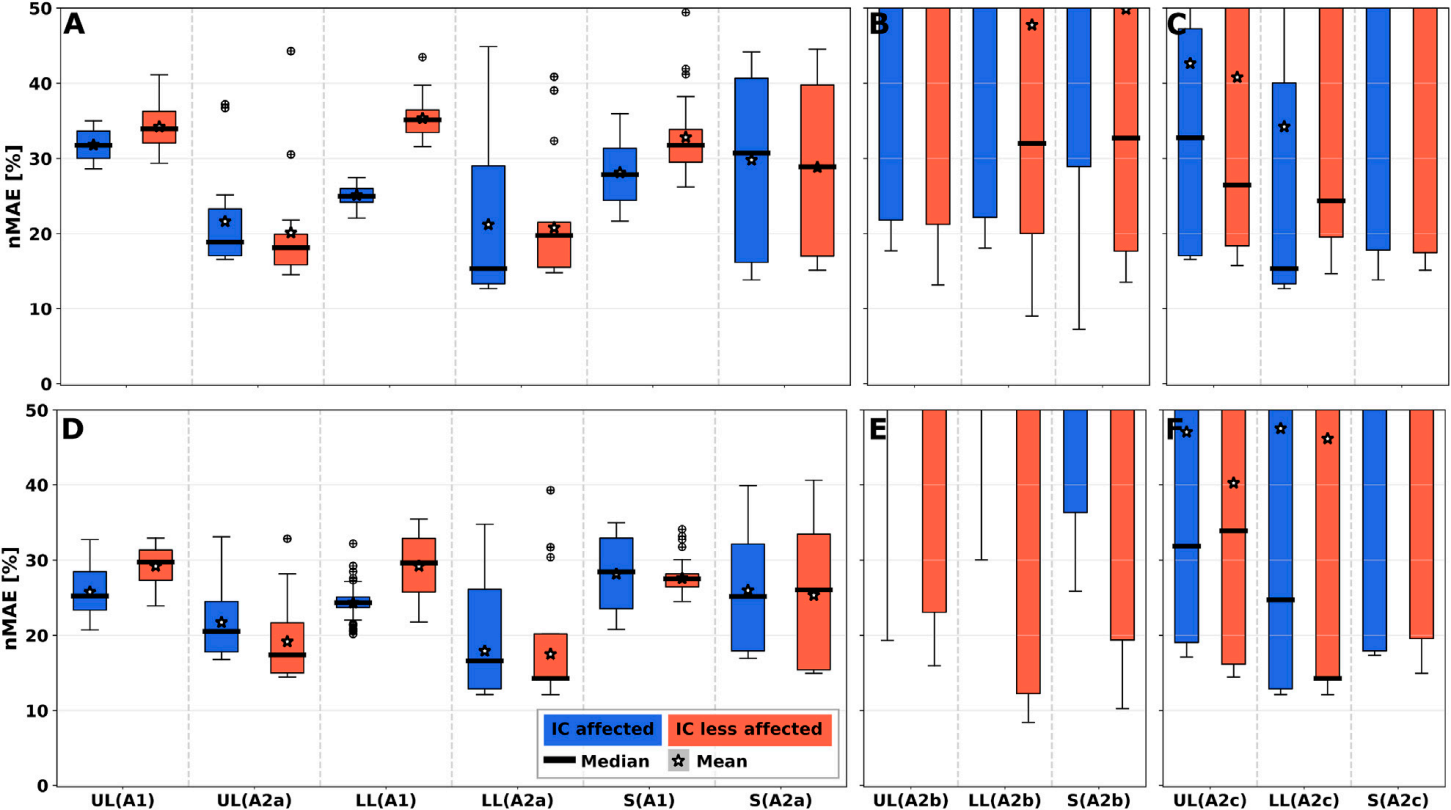
1-phase gait event nMAE of all simulated sensor positions summarised per body segment for all algorithms. nMAE axes were limited to highlight practically relevant sensor performances with nMAE below 50%. For full-range diagrams, please see Supplementary Figure S5. A1 shows larger nMAE in medians for all segments. **(A–C)** PRE intervention. **(D–F)** POST intervention. **(A,D)** User-dependent and sensor position-dependent parameterisation (Algo. A1, Algo A2a). **(B,E)** User-independent parameterisation by averaging parameters across training set patients (Algo. A2b). **(C,D)** User-dependent and sensor position-independent parameterisation by averaging parameters across body segments (Algo. A2c). UL: Upper leg; LL: Lower leg; S: Shoe.

Figure 5 shows the nMAE between ground truth and predicted gait events of all simulated sensor positions on both body sides PRE intervention (see Figures 5A–C) and POST intervention (see Figures 5D–F). Event detection error ranged between 10% and 50%, corresponding to approx. 140–700 ms, depending on the body segment. Algo. A1 showed larger median nMAEs for the upper and lower leg, but lower IQR compared to Algo. A2a. Overall, median nMAE decreased from PRE to POST by 5%–10% on average.

Figures 5B, E show nMAEs of user-independent parameterisation of Algo. A2b, i.e., by averaging parameters across training set patients. Figures 5C, F show nMAEs of user-dependent and sensor position-independent parameterisation of Algo. A2c, i.e., by averaging parameters across body segments. User-indepedency or sensor position-independency increased nMAE median and IQR. Generally, nMAE median improved by about 65% depending on the body segment and algorithm choice (see Supplementary Table S2). Performance degradation is a result of the substantially increased nMAE IQR across sensor positions for Algo. A2b and Algo. A2c. Still, selected sensor positions (e.g., S5/S13 on upper and lower legs or S9/S15 on R3 on the shoe) could be identified that exhibit similar performance as the personalised algorithm variant (A2a), which can be seen in Supplementary Figure S8.

nMAE larger than 100% appeared due to missing peaks in at least one stride, or detecting additional peaks, which resulted in increased event time differences with respect to ground truth. Supplementary Figure S12 illustrates detection errors for two selected sensor positions.

**FIGURE 6:**
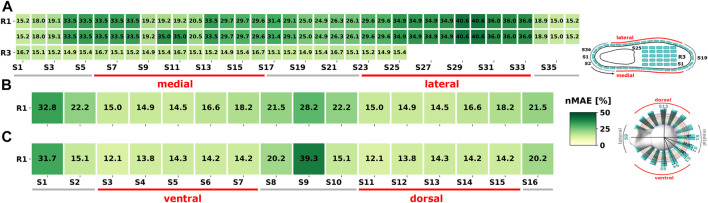
1-phase model: Exemplary nMAE heatmaps POST intervention on the less-affected body side for Algo. A2a. **(A)** All simulated shoe sensor positions. **(B)** Selected sensor ring at the upper leg. **(C)** Selected sensor ring at the lower leg. R: Ring; S: Sensor.

Shoe sensor positions showed larger error IQRs compared to upper and lower legs (see Figure 5), which can be attributed to varying orientation of the shoe-dependent sensors (see also Figure 3). Shoe sensor heatmaps showed a MAE decrease by ∼ 50% for instep and heel sensor positions compared to the shoe positions on the lateral and medial side (see Figure 6). Upper and lower leg positions present circular error patterns as gyroscope-based detection methods, i.e., Algo. A2, do not depend on limb axes shifts. Therefore, Figures 6B, C show one selected sensor ring only, to illustrate the error patterns. Medial and lateral sides of body segments were more prone to errors. The error pattern can be explained by the shift in the main sensor rotation axis during walking. Medial and lateral sensors rotate around an orthogonal axis compared to dorsal and ventral ones. In general, sensor orientation signals are inverted for opposing limb positions, which will affect detection performance for algorithms without position-adapted parameters or models. More detailed error analyses of all body segments can be found in the Supplementary Material.

### 7.2 Event detection with the 2-phase model

**FIGURE 7:**
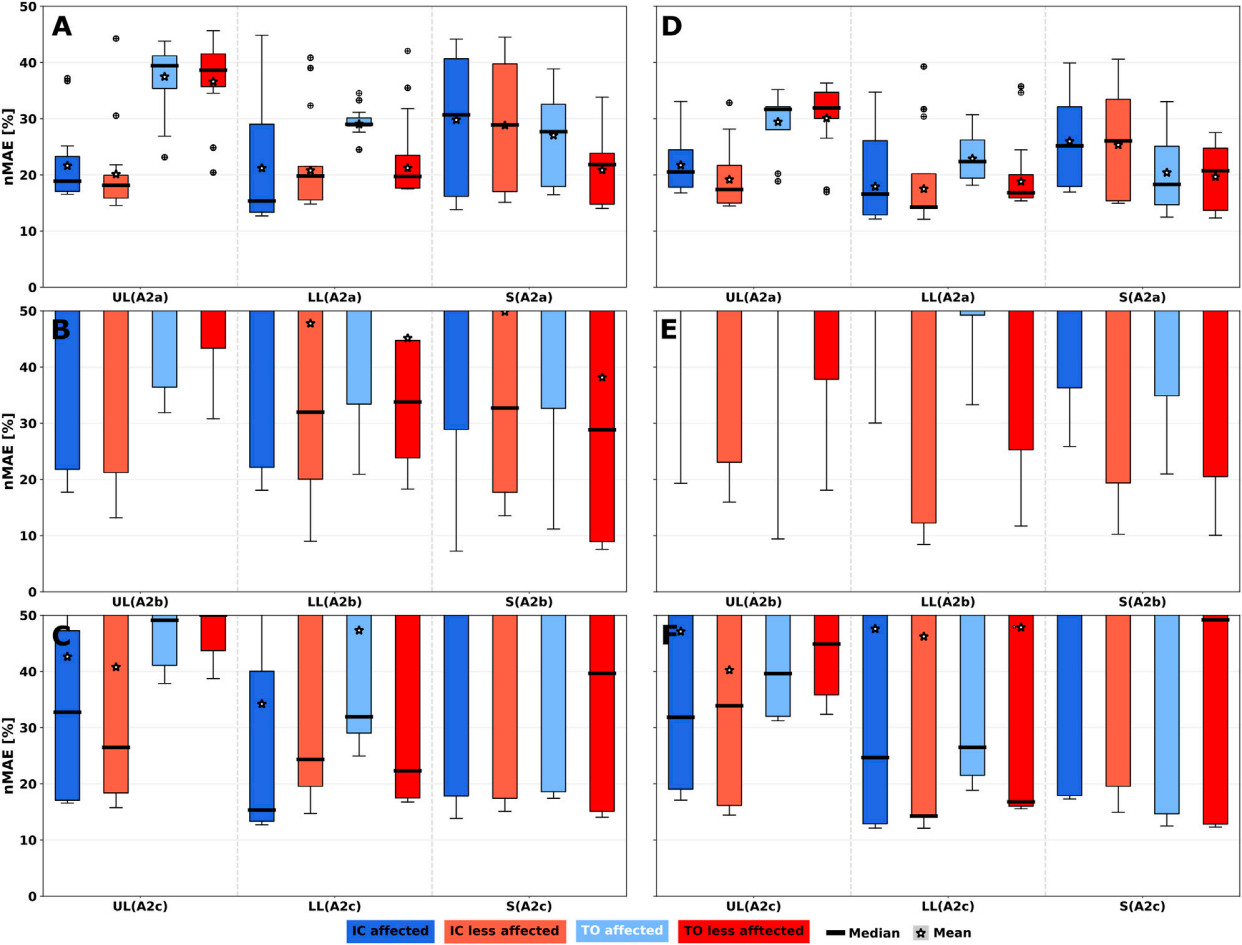
2-phase gait event nMAE of all simulated sensor positions summarised per body segment for Algo. A2 variants. nMAE axes were limited to highlight practically relevant sensor performances with nMAE below 50%. For full-range diagrams, please see Supplementary Figure S6. Gait events are Initial Contact (IC) and Toe off (TO). **(A–C)** PRE intervention. **(D–F)** POST intervention. **(A,D)** User-dependent and sensor position-dependent parameterisation (A2a). **(B,E)** User-independent parameterisation by averaging parameters across training set patients (A2b). **(C,F)** User-dependent and sensor position-independent parameterisation by averaging parameters across body segments (A2c). UL: Upper leg; LL: Lower leg; S: Shoe.

Since our analysis of the event detection with the 1-phase model yielded superior results for gyroscope-based algorithms compared to acceleration only algorithm A1, only Algo. A2 variants (A2a, A2b and A2c) were considered for increased phase granularity. Figure 7 shows nMAE for both predicted gait events of the 2-phase model of all simulated sensor positions on both body sides PRE (see Figures 7A–C) and POST (see Figures 7D–F). Additionally, nMAE for the three algorithm variants are shown. Parameter fitting showed the best performance of all variants (see Figures 7A, D). Consequently, user-independent parameterisation (Algo. A2b) resulted in largest median and IQR for both gait events (see Figures 7B, E; Supplementary Table S3).

TO events showed larger median errors for upper and lower legs compared to IC, but smaller IQR, while shoe sensor position median and IQR were smaller for TO events compared to IC events. The result can be explained by the signal characteristic of synthesised data. As Supplementary Figure S16 illustrates, the negative signal peak is more pronounced on shoe sensor positions compared to the other leg segments and thus events can be recognised more robustly than at the leg.

### 7.3 Event detection with the 4-phase model

**FIGURE 8:**
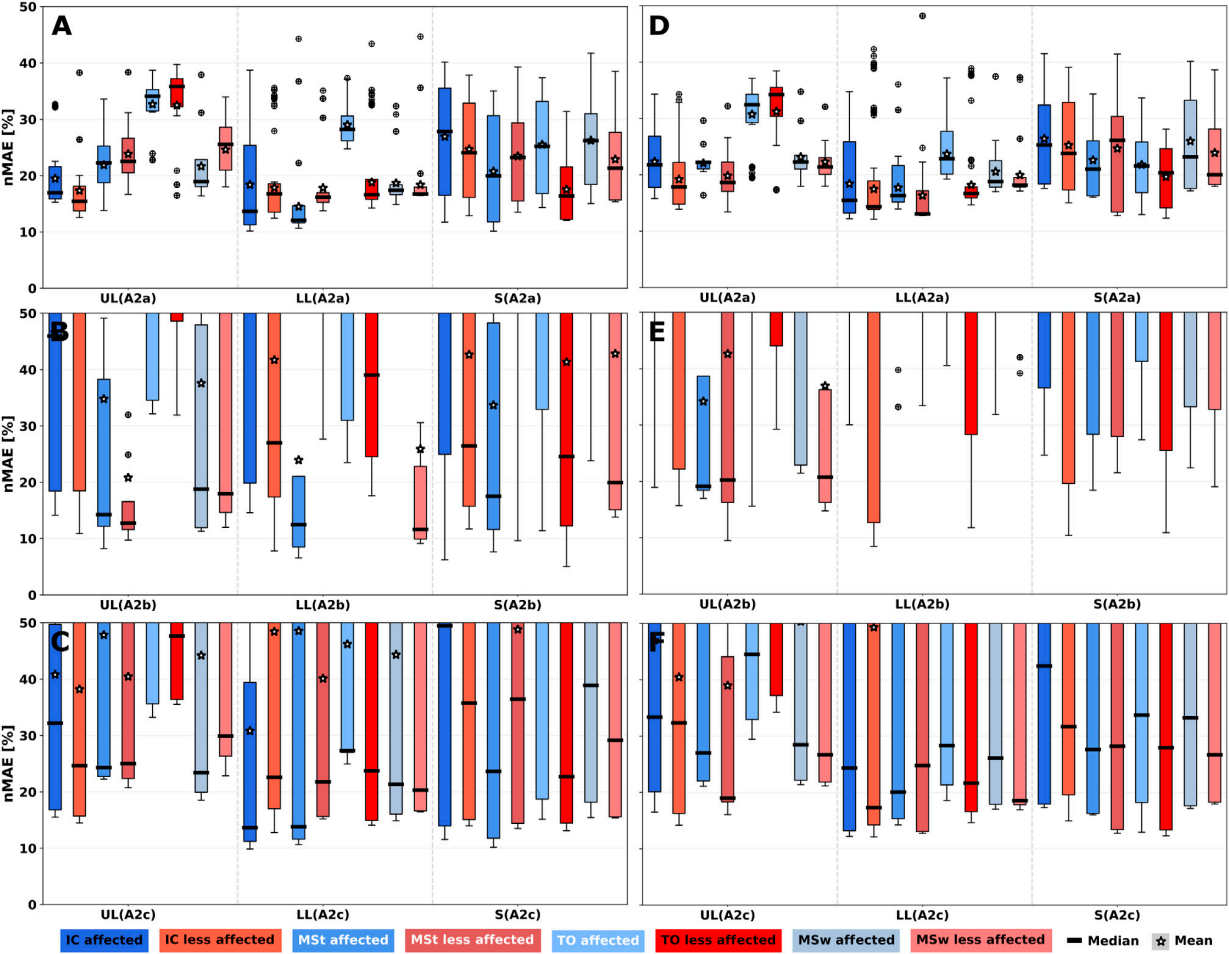
4-phase gait event nMAE of all simulated sensor positions summarised per body segment for Algo. A2 variants. nMAE axes were limited to highlight practically relevant sensor performances with nMAE below 50%. For full-range diagrams, please see Supplementary Figure S7. Gait events are Initial Contact (IC), Mid Stance (MSt), Toe off (TO) and Mid Swing (MSw). **(A–C)** PRE intervention. **(D–F)** POST intervention. **(A,D)** User-dependent and sensor position-dependent parameterisation (A2a). **(B,E)** User-independent parameterisation. **(C,F)** User-dependent and sensor position-independent parameterisation. UL: Upper leg, LL: Lower leg, S: Shoe.

Results of the 4-phase gait event detection are shown in Figure 8. As the phase granularity increases from the 2-phase to the 4-phase model, detection algorithms get more complex with more event dependencies see Sections 4.2.2, 4.2.3, thus resulting in varying estimates for common events among the phase models, e.g., IC. For example, IC event detection yielded 14.3% nMAE in the 2-phase model vs. 17.3% in the 4-phase model and for TO, we observed 16.8% vs. 21.7%, all on the less-affected lower leg using Algo. A2c (see Supplementary Tables S3, S4). IQRs mostly decreased for POST intervention, which is particularly evident at shoe sensor positions. On average, TO showed larger median nMAE at UL and LL for both body sides compared to the other gait events. Among Algo. A2 variants, user-dependent and sensor position-dependent parameterisation (A2a) improved nMAE median and IQR considerably for all gait events of the 4-phase model. User-independent parameterisation of Algo. A2b (see Figures 8B, E) resulted in the largest medians and IQRs for all gait events, indicating that a homogeneous patient group is beneficial when relying on patient-adapted parameters $\left(\theta_{h}\left(\vec{k_{P}}\right)\right)$ . User-dependent and sensor position-independent parameterisation of Algo. A2c (see Figures 8C, F) resulted in a performance deterioration, although less severe than when removing patient adaptation (see also Supplementary Table S4).

### 7.4 Rotatory and translatory errors

**FIGURE 9:**
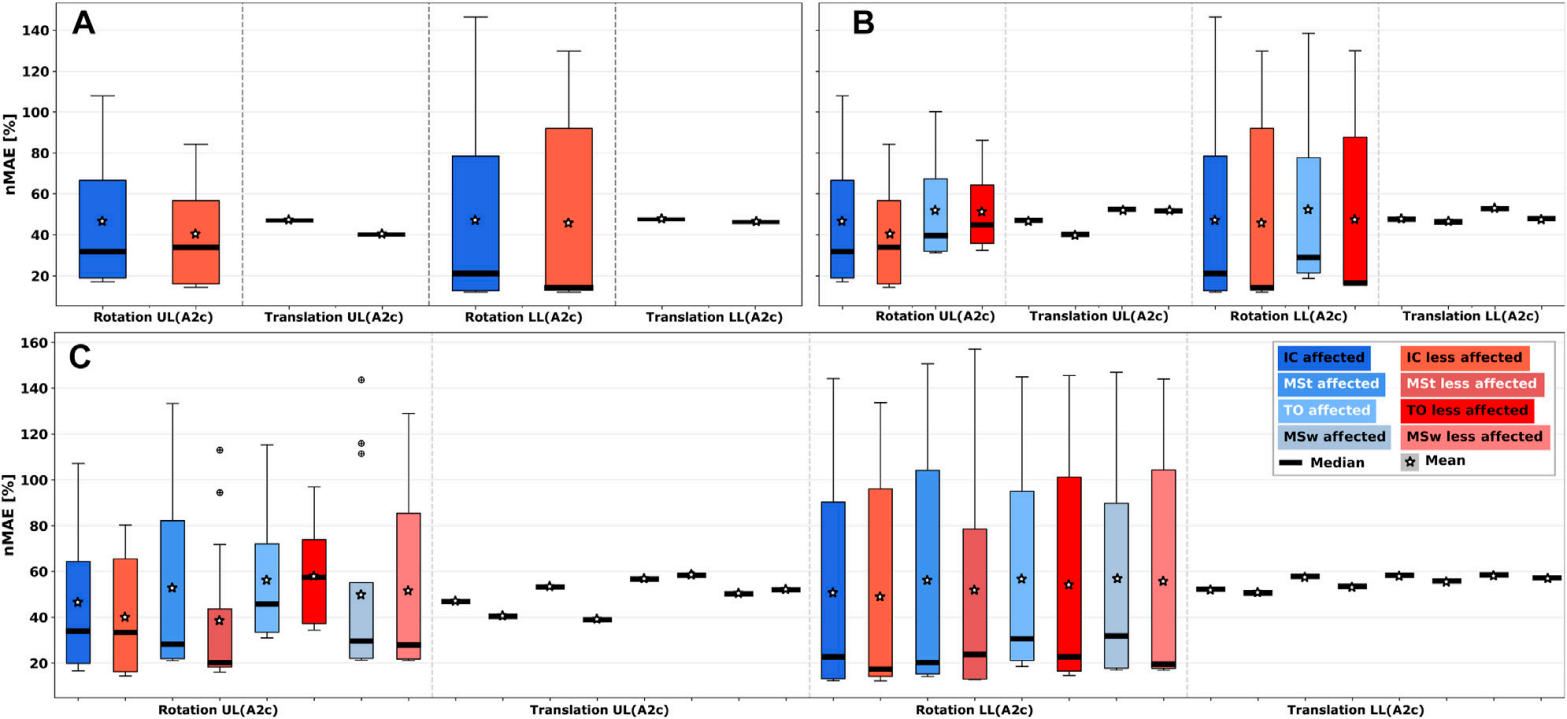
Sensor repositioning error analysis considering rotatory and translatory errors parts for Algo. A2c in all gait phase models. **(A)** 1-phase model. **(B)** 2-phase model. **(C)** 4-phase model. UL: Upper leg; LL: Lower leg. Larger IQRs for rotatory errors can be observed compared to translatory errors, as well as larger error IQRs in the 4-phase model compared to 1- and 2-phase models.

Gyroscope-based algorithms, i.e., Algo. A2, are more prone to rotatory error when repositioning around the axis of the body segment than along the limb axis. Consequently, larger IQRs of rotatory errors can be observed in Figure 9 for all phase models. Due to the translatory data invariance of gyroscope sensors along a body segment, translatory rearrangement along a limb axis did not result in IQRs, and median error remains constant. Shoe sensor positions were excluded from the analysis due to their asymmetric distribution.In 2- and 4-phase models (see Figures 9B, C), TO events for both, affected and less-affected body sides, showed larger median error compared to all other gait events. Increased IQRs for detected gait events can be attributed to a sensor rotation around a body segment. Compared to upper legs, a more uniformly distributed but larger IQR was observed at lower legs, even though error minima were smaller.

When comparing gait phase models, event detection for the 4-phase model showed a subtle increase in rotatory errors for IC and TO events, which could be attributed to the increased algorithm complexity and dependency of gait events compared to the other two phase models. Furthermore, the 4-phase model showed a more uniform error distribution, but larger IQR compared to 1- and 2-phase models. Thus, with increasing phase granularity, error IQRs across sensor positions increased, while the minimum error remained almost constant.

## 8 Discussion

We introduced a comprehensive co-simulation framework to model body motion, i.e., gait patterns of hemiparetic patients, and inertial sensors, to interpret position and algorithm-dependent performance for 1-, 2-, and 4-phase gait event detection. In addition, we have investigated performance depending on rotatory and translatory changes in all three gait phase models to demonstrate versatility of our co-simulation framework.

### 8.1 Model validation

Our comparative model validation (Section 6) aimed at quantifying similarities between synthesised and measured sensor signal patterns, as well as to confirm matching value ranges for acceleration and angular velocity signals. Model validation showed that (1) simulated sensors can approximate healthy walking, and 2) synthesised sensor signals showed moderate correlations (*r* = 0.63) for acceleration signals and high correlations (*r* = 0.95) for angular velocity signals, with nMAD below 11% for both.

Simulations are unlikely to yield perfect agreement with measured inertial sensor signals due to non-modelled, dynamic phenomena, e.g., IMU placement and fixation variability and marker loss. Nevertheless, our results across all sensor signals suggested meaningful synthesis performance. We attributed deviations for acceleration at lower legs and feet (see Figures 4B, C) to positioning variability along the gravitational axis that could naturally occur for sensor mounts and were not adequately captured by our sensor model’s auxiliary structures. RMSE and correlations observed in our validation analysis are within ranges reported in literature. Sharifi Renani et al. (2021) compared synthetic and measured IMU data at the pelvis, thigh, shank, and foot during walking, where RMSE for angular velocity ranged between 22.9°/s - 58.4°/s depending on sensor positioning. Correlations ranged between r = 0.3 – 0.9. RMSE for acceleration ranged between 0.6 m/s^2^ - 2.5 m/s^2^ with correlations between r = 0.7–0.9. Zimmermann et al. (2018) reported average correlations of r = 0.6 for accelerometers and r = 0.9 for gyroscopes at feet, shanks, thighs, and pelvis during walking. Average RMSE was 4.0 m/s^2^ and 35°/s for acceleration and angular velocity, respectively. The authors attributed the synthetic gap to additional artifacts due to clothing and soft-tissue that cause additional accelerations. Future work may focus on natural body shape representation by surface modeling, which can decrease model deviations (Uhlenberg and Amft, 2022).

### 8.2 Algorithm and performance analysis

For the present analysis, we selected parameterisable online, i.e., sliding window-based algorithms that could operate in real-time. The selected algorithm family extracts gait cycle events after detecting an initial contact following the respective gait cycle. Hence, the algorithms incur an event reporting delay of approx. one gait cycle. Algorithm parameters were initially fitted in a cross-validation procedure, but remained constant during the evaluation. Algo. A2a represents a typical personalised parameterisation, but implies that an initial parameter fitting is made, based on user-specific data. Algo. A2b was user-independent by fitting parameters from population data and thus might be easier deployed. Finally, for Algo. A2c sensor position dependency was removed, hence training data could originate from elsewhere at the same limb. The analysed algorithms are widely applied for gait event detection. Nevertheless, our co-simulation framework is not limited to one algorithm family. Other gait event detection algorithms, e.g., based on Hidden-Markov Models (HMM), as well as extended gait phase models, e.g., by additional gait phases, can be analysed using the proposed framework. Algorithms considered in our analysis rely on a bipedal detection approach, adapted from Zhao et al. (Zhao et al., 2019), which may be debatable with regard to body side-dependent gait patterns of hemiparetic patients. Our evaluation results however show that using the contralateral side to support gait event detection did not influence detection error of MSt and MSw events. The distribution of recognition errors in the 4-phase model was relatively constant across all phases. Similarly, the estimation errors of IC and TO in the 4-phase model were comparable to those of the 2-phase model (see Supplementary Tables S3, S4), even though the detection algorithms get more complex with additional gait events and gait phase granularity. Thus, we encourage further investigations of bipedal detection algorithms in hemiparetic patients.

Results showed better performance of Algo. A2a compared to Algo. A1 for event detection of the 1-phase model, which agrees well with previous measurement studies, e.g., (Lau and Tong, 2008; Kotiadis et al., 2010). A primary explanation of detection performance differences is that angular velocity signals used by A2 algorithms show more periodic and repeatable patterns along the gait cycle than acceleration signals, which confirms previous observations, e.g., made by Taborri et al. (2016).

Among Algo. A2 variants, user-dependent and sensor position-dependent parameterisation (Algo. A2a) showed lowest nMAE median (13.1%–41.4%) and IQR (1.4%–25.6%) for all gait events and gait models. User-dependent and sensor position-independent parameterisation (Algo. A2c) led to performance deterioration (increase in median nMAE by 5.5%–19.5%), although less severe than user-independent parameterisation (Algo. A2b), which showed an increase in median nMAE by 24.9%–80.2%. Parameter personalisation clearly adds algorithm complexity. Consequently, individual gait pattern variability should be specifically considered in future studies of gait event detection algorithms, e.g., by comparing algorithms under gait and sensor positioning variations and incorporating algorithm personalisation methods, where necessary. Most gait detection investigations to date have focused on healthy volunteers, fixed sensor position, and algorithm parameterisation with manually adjusted parameters or once-trained statistical models (Catalfamo et al., 2010; Kotiadis et al., 2010; Rueterbories et al., 2014; Maqbool et al., 2016; Sánchez Manchola et al., 2019). Our results however show that for hemiparetic patients a major potential to minimise event detection errors lies in adapting (e.g., personalising) the main rotation axis at medial-lateral vs. dorsal-ventral, depending on sensor position. We achieved the intended user-dependency by splitting the per-participant synthesised timeseries into folds. User-dependency is generally considered to maximise detection performance, but risking generalisability. Due to the 5-fold CV splits of the timeseries, testing data may be overly dependent on training data. Nevertheless, personalisation of different gait detection models has recently been successfully applied for other neurological disorders and gait rehabilitation robots (Martindale et al., 2018; Ye et al., 2020; Ingelse et al., 2022). Based on our results, further work on algorithm personalisation is warranted. Furthermore, we observed that particular sensor positions, e.g., at the shoe, show consistently robust signal patterns suggesting minimal detection errors (see Supplementary Figure S11, S16). Sensor positions found with our co-simulation framework may be favourable for gait event detection or serve as an alternative reference position in measurement studies, where minimal instrumentation is required.

nMAE median and IQR decreased on all body segments from PRE to POST intervention for all phase models, which could indicate an improvement in gait regularity at POST intervention. The hypothesised gait improvement is supported by findings of Knarr et al. (2013) and Derungs and Amft (2020), who respectively showed increased muscle activation of plantar flexors and decreased stride duration after rehabilitation intervention for the same dataset.

To compare our results with those of Derungs and Amft (2020), we additionally performed an analysis of stride duration using the normalised root mean square error (nRMSE) as described in the Supplementary Equation S2 and Supplementary Figure S5. However, the aforementioned work analysed upper leg sensor positions and dynamic acceleration only. Furthermore, ground truth data was taken from the heel MoCap marker rather than GRF in our work, which limits the comparison. Compared to Algo. A1 in our work; Derungs and Amft (2020) showed smaller nRMSE for the best performing sensor (0% vs. 17%), but larger errors for the worst performing sensor (44% vs. 36%), which may be due to the combined static and dynamic acceleration model and GRF-based ground truth used in our work. Algo. A2 showed similar nRMSE for the best sensor (0.1%) and considerably smaller errors for the worst performing sensor (8%). Thus Algo. A2 improves the error bound compared to an acceleration-based detection.

Timing error across hemiparetic patient dataset varied considerably between algorithm variants and sensor positions, ranging from 16% to 160% mean nMAEs, corresponding to temporal event deviations of approximately 220 ms–2,240 ms. Timing errors reported by previous measurement studies ranged between −28 ms and +190 ms, e.g., (Behboodi et al., 2015; Chia Bejarano et al., 2015; Gouwanda and Gopalai, 2015; Zhou et al., 2016; Lambrecht et al., 2017; Sánchez Manchola et al., 2019). We assume that elevated error ranges may be observed for particular patient groups and specific study designs (e.g., PRE and POST intervention). However, a substantial share of the larger error ranges found in our simulations can be explained by utilising an absolute error metric, instead of averaging signed errors. When averaging individual signed errors, early and late event detection errors across individual gait cycles may compensate and thus, result in artificially smaller mean error reporting. Similarly, metrics derived from signed error, including accuracy, precision, and recall may conceal the actual event timing error. Therefore, we suggest that MAE or a metric variant relative to stride duration, i.e., nMAE, as defined in Eq. 16, should be applied for analyses of sensor positioning and algorithm variants.

### 8.3 Biomechanical body model and shoe model

We consider our approach robust and reproducible: we used previously applied and validated biomechanical models in the well-established and validated musculoskeletal modeling environment OpenSim for our analysis (Delp et al., 2007; Seth et al., 2018; Karimi et al., 2021; Yap et al., 2021). We applied default personalisation procedures, using participant body weight and MoCap markers with unchanged default weights. Our sensor models were derived using fundamental physics-based equations. We validated our co-simulation approach by comparing synthesised data and actual IMU measurements (i.e., IMU data without modeling biases), resulting in meaningful synthesis performance. Nevertheless, further work may include sensitivity analyses, e.g., on MoCap marker weights and further algorithm parameters, to explore simulation variability.

According to rigid-body dynamics, vertical sensor translation along a body segment does neither affect rotation nor angular velocity. Thus, with a symmetric sensor distribution around limb axes, synthesised sensor signals and timing errors are identical at each ring of upper and lower legs (e.g., S1 on R1 vs. S1 on R2, see Figure 3.). Our error analysis revealed that A2 algorithms were more likely to incur rotational errors than translational errors, which is a direct effect of gyroscope-based measurement. To add further co-simulation fidelity, sensor placement may follow natural body shapes more accurately, e.g., captured from silhouette data. Further biomechanical research is needed to adapt silhouette modeling concepts, e.g., those currently investigated for animation purposes (Loper et al., 2015). A particular challenge of animation models is to maintain anatomically correct representation during motion (Baronetto et al., 2021). We deployed non-deformable, feature-less geometric silhouette models for shoes, which may insufficiently represent natural sensor signal variation. Similar to the auxiliary structure at limbs, the shoe model served as a substrate for sensors and was not individually validated. Shoe design and mechanical properties vary, including materials, fixtures, and rigid structures. We believe that our co-simulation framework enables researchers to investigate design variations by adding corresponding mesh models into the simulation, e.g., shoe and cloth designs.

A direct-link weld joint was used to simulate sensor attachment. While a fixed sensor-bone fixation may not be practically useful, it enabled us to contrast placement and algorithm parameterisations in the present analysis, which emphasised relative differences. Derungs and Amft (2020) showed that simulating accelerometer attachment variation by adding sinusoidal signal noise had only minor effects ( < 5% change of nRMSE for step duration detection) (Chèze et al., 1995). Similarly, Bogaarts et al. (2021) showed that for a simulated smartphone worn at the pelvis, adding Gaussian white noise *via* Monte Carlo Simulation, had a negligible impact on estimating step power. We believe that future research on realistic attachment modeling is needed to study sensor performance effects and that our co-simulation framework can provide an appropriate environment. While we already show how non-ideal sensor placement influences sensor readings (e.g., see Supplementary Figure S13–S15), further work should simulate sensor specification variants to investigate operational limits, including signal noise and gyroscope bias.

### 8.4 Perspectives

The proposed co-simulation framework focuses on optimising information retrieval. For a wearable system realisation, other sensor positioning priorities, e.g., according to user sensor wearing preferences and technical feasibility, could be considered. However, we consider that our information-driven simulation framework can provide system designers with an initial priorisation to select sensor positions, algorithms, and parameters. Furthermore, co-simulations enable us to represent temporal dynamics of individual motion and compensation patterns that are challenging to describe analytically.

Our methodology enables wearable system designers and algorithm developers to find suitable sensor types, position, and algorithm parametrisation, when MoCap data, or similar body orientation data is available. To implement an arbitrary algorithm analysis, developers may follow the methods described in Sections 3, 5.

Results of our analysis confirm a new pathway for wearable system development and *in silico* performance evaluation using human digital twins. Dynamic, model-based simulations of on-body systems could be used as a preliminary assessment before testing in the physical world. For example, our co-simulation framework could be used to assess wearable inertial sensor systems for clinical testing, athletic tracking, and further proof of concepts. In addition, initial simulation-based exploration could help in planning of remote monitoring applications, before physical prototypes are deployed or even fabricated. The human digital twins developed in this work describe biomechanics and motion phenomena, not any underlying neurological deficits. However, neural control affects kinematics, which were captured in the included study data.

## 9 Conclusion

We introduce a co-simulation framework that explores how personalised biomechanical and motion sensor models can assist in gait detection algorithm analysis and wearable system design to achieve optimal body placement. Initially, we validated sensor signal synthesis in a qualitative comparison against measurements at the same participants, confirming suitable performance of our co-simulation approach. Subsequently, we evaluated common gait event detection algorithms within our co-simulation framework and showed absolute detection errors.

Gait phase analysis in hemiparetic patients demonstrated that our framework can deal with highly complex gait patterns. The best gait event detection performance was observed for an user-dependent and sensor position-dependent algorithm parameterisation (Algo. A2a), an increase of 65% compared to an algorithm with user-independent parameterisation (Algo. A2b). For comparison, sensor-position dependent parameterisation added only 25% compared to position-independent detection (Algo. A2c). While our simulations confirmed that gyroscope-based gait event detection algorithms outperformed their acceleration-based counterparts, the rotatory sensor error analysis demonstrated limitations of a gyroscope-based gait phase detection: We found larger IQRs when repositioning sensors around the main body segment axis compared to repositioning sensors along the limb axis for the gyroscope-based detection (Algo. A2c). We showed that our framework can be used to evaluate different algorithms as well as different gait phase models. For the commonly used algorithms considered in this work, our analysis showed that there is a larger dependency on the user than sensor position. Thus, user personalisation of gait phase detection should be considered, while sensor position variation may be a secondary adaptation target.

Our co-simulation framework allowed us to evaluate arbitrary sensor positions on the body. For example, sensor positions at the shoe can lead to larger errors compared to upper and lower legs. In addition, we can evaluate specific geometries, including shoe shape, to determine optimal placement. For the shoe, instep and heel positions were more suitable compared to lateral and medial sensor positions.

Our approach opens a new pathway for utilising dynamic human digital twins in wearable system design and performance estimation, before physical prototypes are deployed or manufactured.

## Data Availability

Publicly available datasets were analyzed in this study. This data can be found here: https://simtk.org/projects/fesprediction.
