# Supplementary material for: Co-simulation of human digital twins and wearable inertial sensors to analyse gait event estimation
Source: Front Bioeng Biotechnol. 2023 Apr 12;11:1104000. doi: 10.3389/fbioe.2023.1104000 (PMC10132030; doi:10.3389/fbioe.2023.1104000)
Supplement: Supplementary file 1 [file DataSheet1.pdf]

Co-Simulation of Human Digital Twins and Wearable Inertial Sensors to Analyse Gait Event Estimation

Lena Uhlenberg [1*,2], Adrian Derungs [3] and Oliver Amft [1,2]

[1] Hahn-Schickard, Freiburg, Germany
[2] Intelligent Embedded Systems Lab, University of Freiburg, Freiburg, Germany
[3] F. Hoffmann–La Roche Ltd, pRED, Roche Innovation Center Basel, Basel, Switzerland

Coresspondance*:
Lena Uhlenberg
lena.uhlenberg@hahn-schickard.de

1 SUPPLEMENTARY MATERIAL

This document provides supplemental detail to the paper: Uhlenberg L, Derungs A and Amft O (2023), Co-simulation of human digital twins and wearable inertial sensors to analyse gait event estimation. Front. Bioeng. Biotechnol. 11:1104000. doi: 10.3389/fbioe.2023.1104000

For model validation measurements, Tab. S1 shows participant details and Fig. S1 shows our study setup.

Table S1. Anthropometric data of participants. F: Female. M: Male. BMI: Body mass index.

**ID**	**height [m]**	**weight [kg]**	**age [years]**	**sex [m/f]**	**BMI [kg/m2]**
**P1**	1.80	74	25	M	22.84
**P2**	1.71	56	23	F	19.15
**P3**	1.78	65	29	M	20.52
**P4**	1.83	79	25	M	23.59
**P5**	1.68	68	36	F	24.09
**P6**	1.69	62	24	W	21.71
**P7**	1.70	65	29	M	22.49
**P8**	1.65	62	28	F	22.77
**P9**	1.73	66	22	F	22.05
**P10**	1.45	48	21	F	22.83
**Mean**	**1.70**	**63.96**	**25.89**	**F=4; M=8**	**22.20**
**STD**	**0.10**	**8.20**	**4.21**		**1.38**

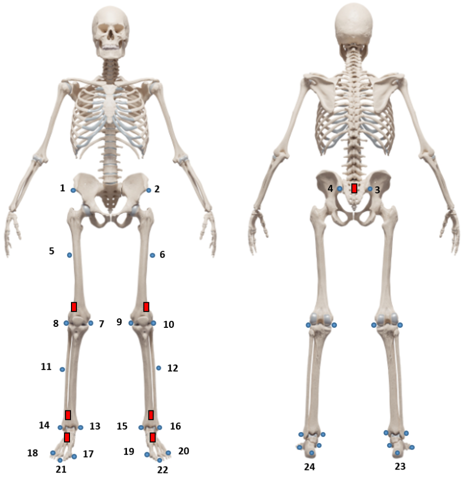

Figure S1. Model validation setup. Six IMUs and 24 MoCap markers were attached to the lower limbs of ten healthy participants.

Stride duration estimation differs from gait event detection, as it does not consider whether gait events are correctly identified along the time axis, i.e. stride duration estimation reports a relative time between IC events, thus does not consider absolute time differences between detected events $\hat{t}_i$ and reference events t_i (see Eq. 7 in the main publication). Stride duration estimation accuracy was determined by deriving the mean absolute error (MAE) across all U_s strides per test fold according to:

$$MAE = \frac{1}{U_s} \sum_{i=1}^{U_s} \left| (t_i - t_{i-1}) - (\hat{t}_i - \hat{t}_{i-1}) \right| , \tag{S1}$$

with $\hat{t}_i$ as detected IC events and t_i as reference IC events. Subsequently, normalised MAE (nMAE) was obtained in percent by normalisation with the reference average stride duration $\overline{F}$ per body side, intervention condition, and patient (see Eq.16 in the main publication).

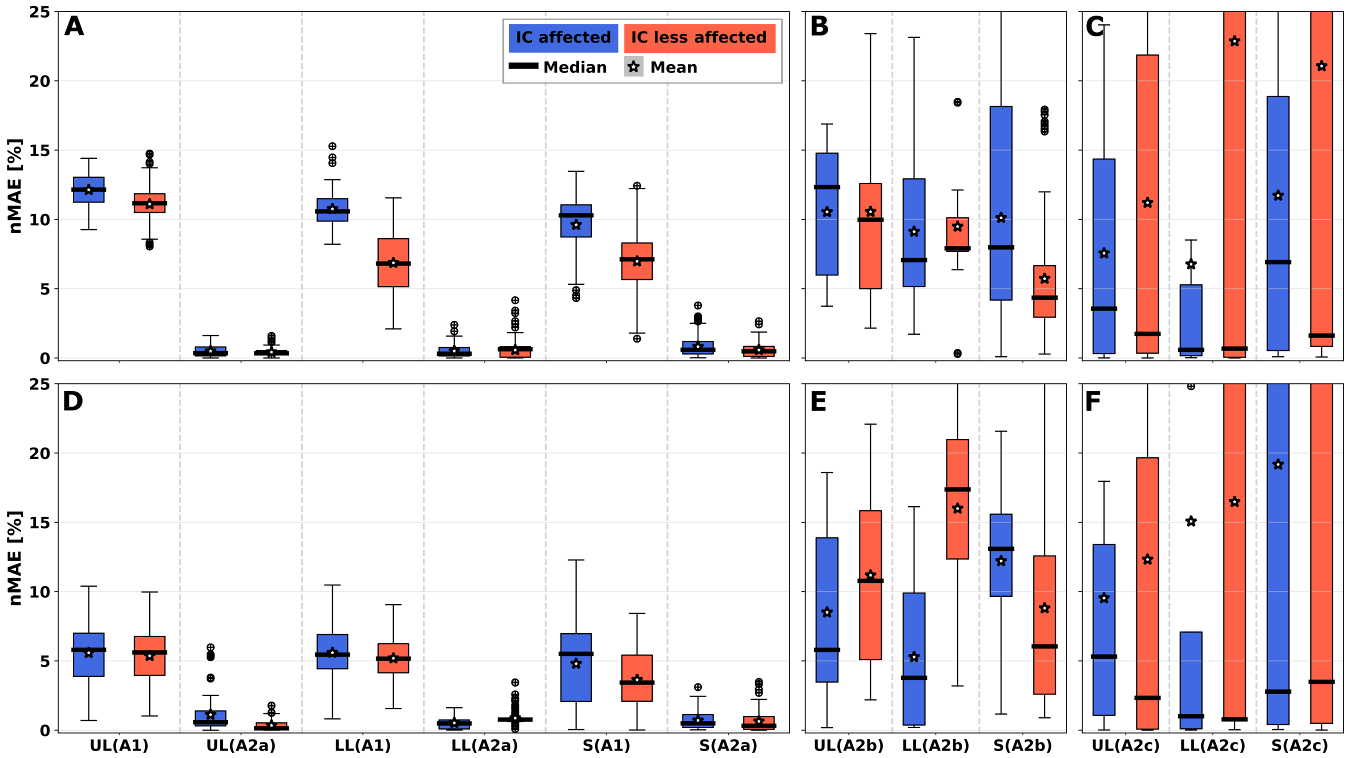

Figure S2. Stride duration nMAE of all simulated sensor positions summarised per body segment for all algorithms. nMAE axes were limited to highlight practically relevant error ranges, resulting in the omission of errors and interquartile ranges in some segments. A1 shows larger nMAE in medians and IQRs compared to A2. A-C: PRE intervention. D-F: POST intervention. A,D: User-dependent and sensor position-dependent parameterisation (Algo. A1, Algo A2a). B,E: user-independent parameterisation by averaging parameters across training set patients (Algo. A2b). C,F: User-dependent and sensor position-independent parameterisation by averaging parameters across body segments (Algo. A2c). UL: Upper leg, LL: Lower leg, S: Shoe.

Figure S2 shows stride duration nMAE of all simulated sensor positions at both body sides PRE intervention (see Fig. S2A-C) and POST intervention (see Fig. S2D-F). On average, nMAE decreased from PRE to POST intervention. Between algorithms, A2a outperformed A1 that uses accelerometer data only (see Fig. S2A,D). Algo. A2a that used gyroscope and accelerometer data showed lower nMAE median and IQR across all sensor positions and body segments. nMAE of Algo. A1 decreased by almost 50% from PRE to POST, while error for A2a decreased only marginally.

Initially, algorithm parameters were fitted to individual patients and sensor positions following the methodology described in Sec 3.5 in the main publication. User-independent parameterisation by averaging parameters across training set patients (Algo. A2b, Fig. S2B,E), or user-dependent and sensor position-independent parameterisation by averaging parameters across body segments (Algo. A2c, Fig. S2C,F) resulted in increased estimation errors. Compared to Fig. S2A and D with user-dependent and sensor position-dependent parameterisation, where nMAE ranged below 5%, removing user-dependency or body segment dependency increased error by 10%-20% on average, depending on the segment and algorithm choice. Hence, when removing parameter fitting, sensor positioning and algorithm-based error compensation is essential. As instep positions are not symmetric around the shoe model (see Fig. Fig. 3 in

main publication), heatmaps comprising all simulated shoe sensor positions on the less-affected body side are shown in Fig. S3. Heatmaps of upper and lower legs, as well as the shoe on the affected body side can be found in Fig. S9- S11.

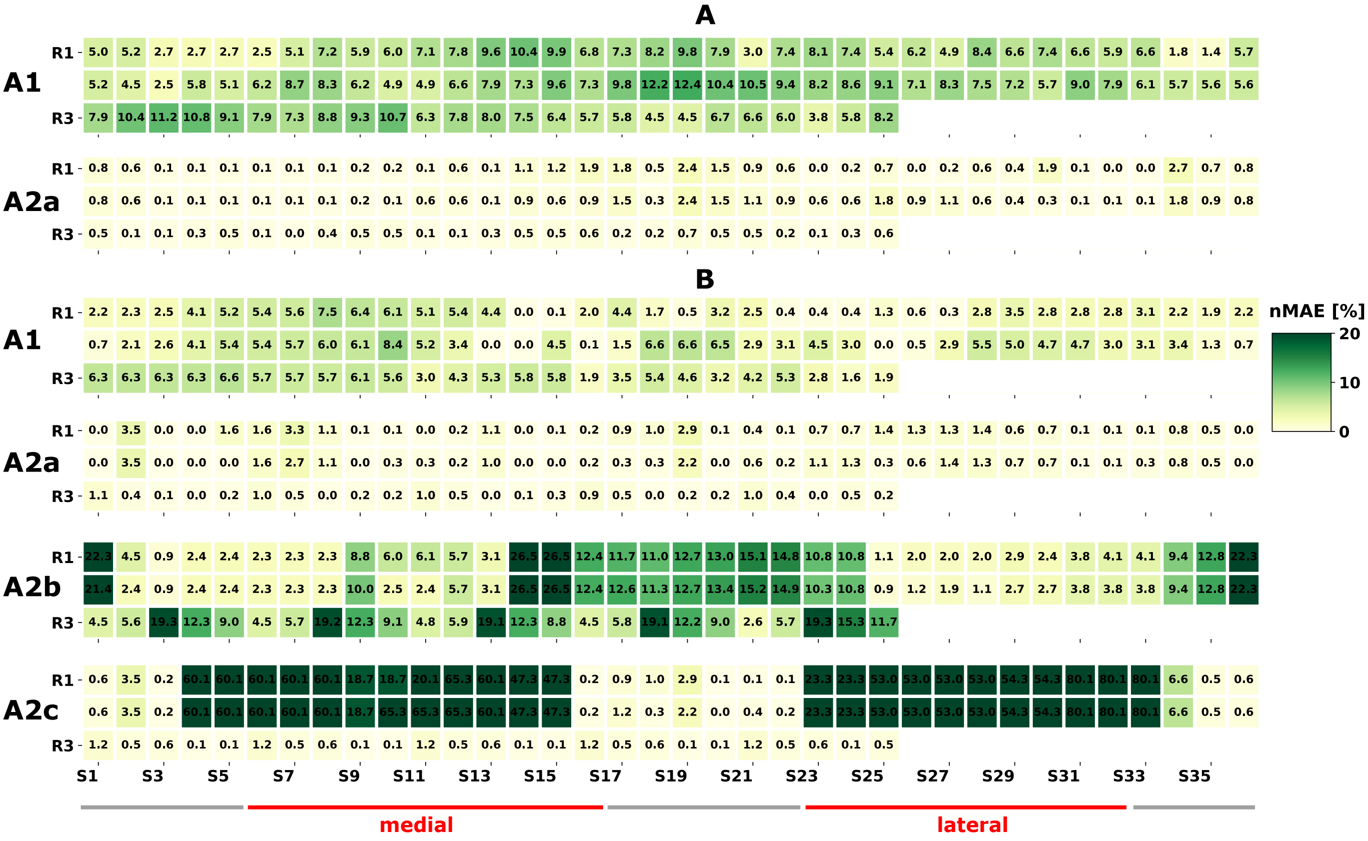

Figure S3. Stride duration nMAE of shoe positions on the less affected body side for accelerometer only Algo. A1 and three variants of Algo. A2a. A1 shows larger nMAE compared to A2. A: PRE intervention. B: POST intervention. A2a: User-dependent and sensor position-dependent parameterisation. A2b: User-independent parameterisation by averaging parameters across training set patients A2c: User-dependent and sensor position-independent parameterisation by averaging parameters across body segments. R: Ring. S: Sensor

On average, nMAE decreased after intervention for both algorithms (see Fig. S3B). Here too, Algo. A2a is superior to A1, showing lower nMAEs across all sensor positions (0.1%-4.4% vs. 0.2%-17.3%). User-dependent and sensor position-independent parameterisation by averaging parameters across body segments increased nMAE on medial and lateral sides for Algo. A2c . For Algo. A2c sensors on shoe tip and instep show lowest nMAE. In contrast, averaging algorithm parameters over training set patients (Algo. A2b) nearly inverted estimation performance.

Figure S4 shows full range stride duration nMAE (see Fig. S2) of all simulated sensor positions at both body sides PRE intervention and POST intervention.

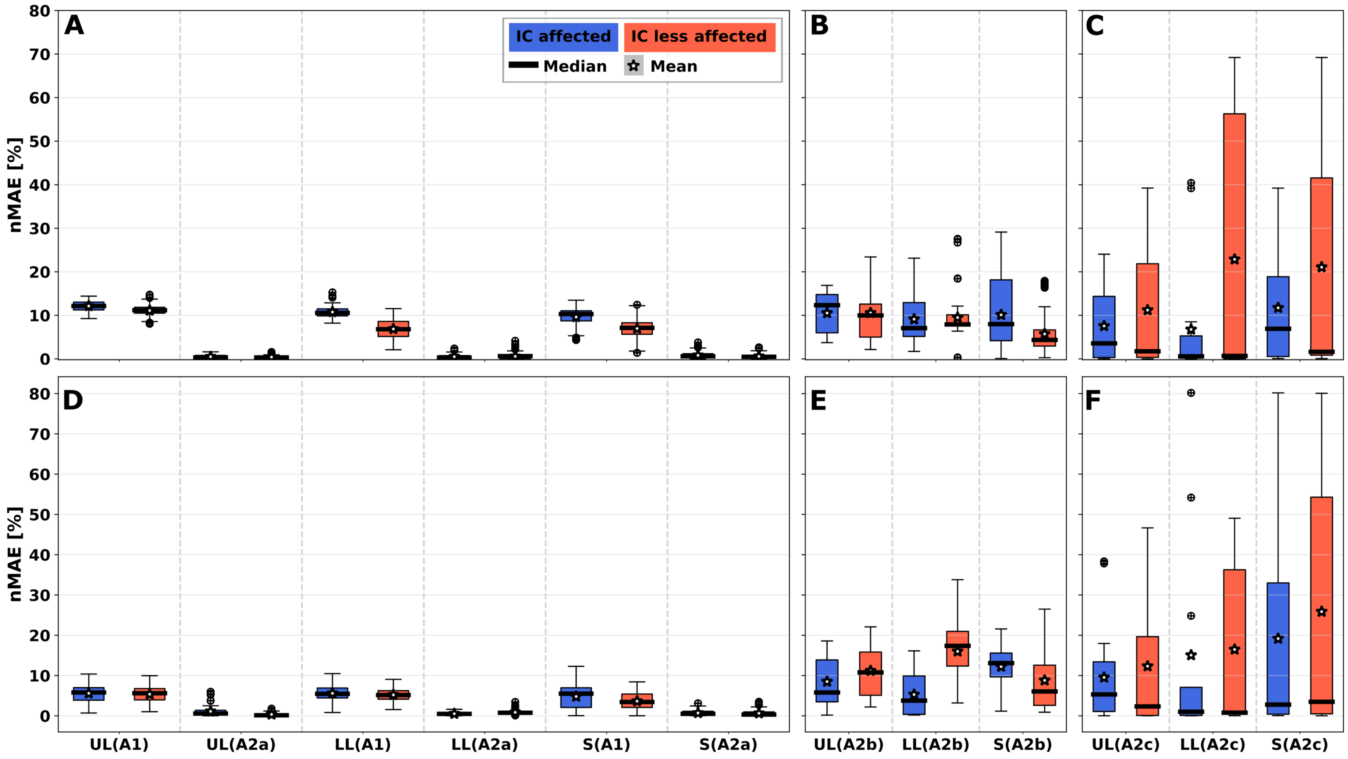

Figure S4. Full range stride duration nMAE of all simulated sensor positions summarised per body segment for all algorithms. A1 shows larger nMAE in medians and IQRs compared to A2. A-C: PRE intervention. D-F: POST intervention. A,D: User-dependent and sensor position-dependent parameterisation (Algo. A1, Algo A2a). B,E: user-independent parameterisation by averaging parameters across training set patients (Algo. A2b). C,F: User-dependent and sensor position-independent parameterisation by averaging parameters across body segments (Algo. A2c). UL: Upper leg, LL: Lower leg, S: Shoe.

Figure. S5 shows the 1-phase full range gait event nMAE (see Fig. 5 in main manuscript)between ground truth and predicted gait events of all simulated sensor positions on both body sides PRE intervention and POST intervention.

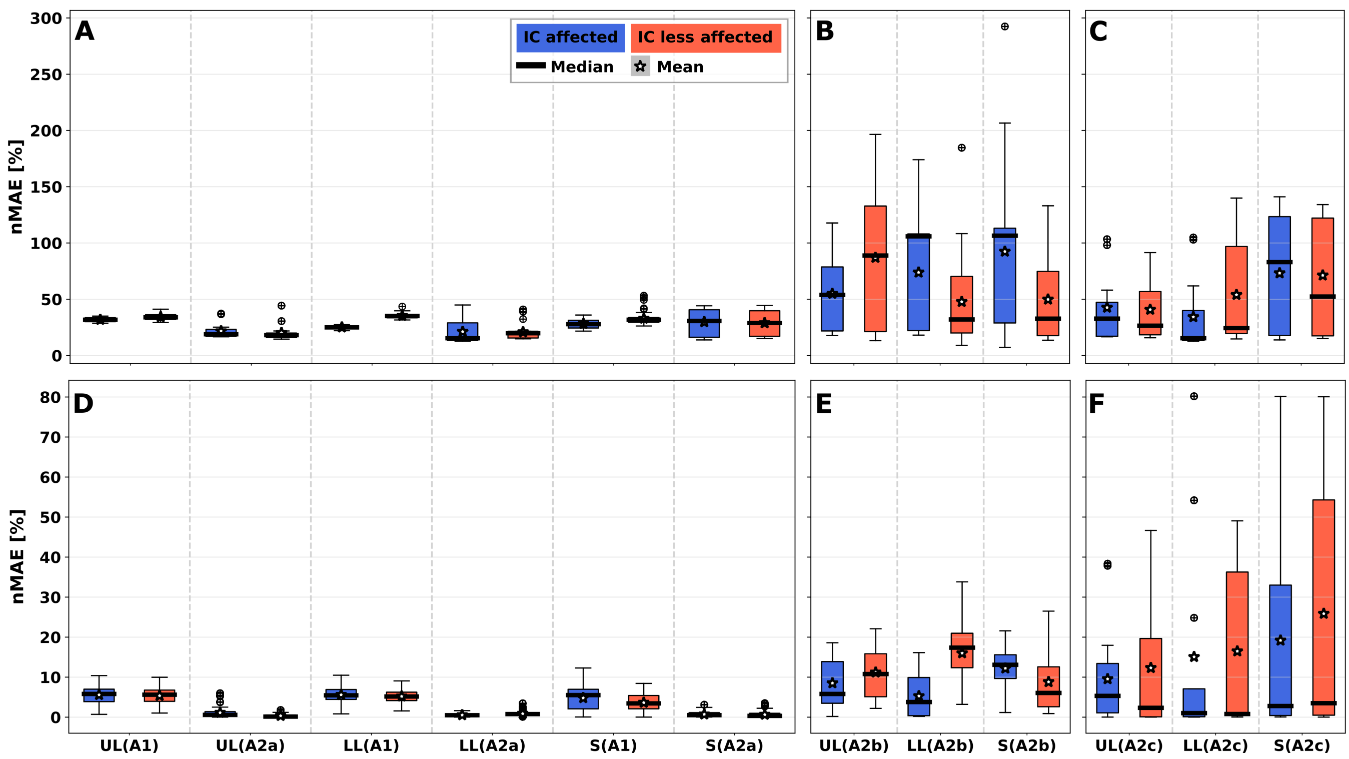

Figure S5. Full range illustration of 1-phase gait event nMAE of all simulated sensor positions summarised per body segment for all algorithms. A1 shows larger nMAE in medians for all segments. A-C: PRE intervention. D-F: POST intervention. A,D: User-dependent and sensor position-dependent parameterisation (Algo. A1, Algo A2a). B,E: User-independent parameterisation by averaging parameters across training set patients (Algo. A2b). C,D: User-dependent and sensor position-independent parameterisation by averaging parameters across body segments (Algo. A2c). UL: Upper leg, LL: Lower leg, S: Shoe.

Figure. S6 shows the 2-phase gait full range event nMAE (see Fig. 7 in main publication) between ground truth and predicted gait events of all simulated sensor positions on both body sides PRE intervention and POST intervention.

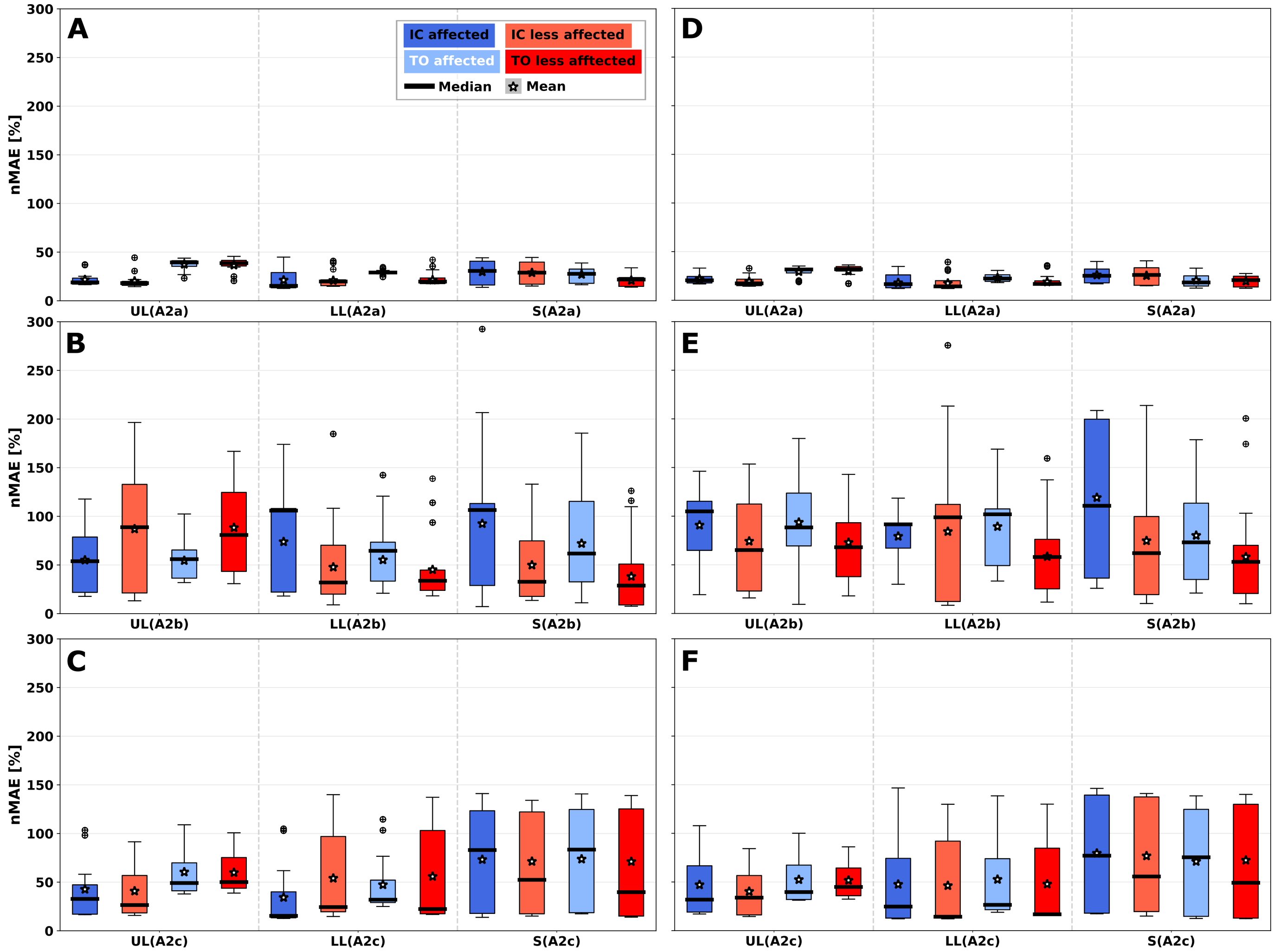

Figure S6. Full range illustration of 2-phase gait event nMAE of all simulated sensor positions summarised per body segment for all algorithms. A1 shows larger nMAE in medians for all segments. A-C: PRE intervention. D-F: POST intervention. A,D: User-dependent and sensor position-dependent parameterisation (Algo. A1, Algo A2a). B,E: User-independent parameterisation by averaging parameters across training set patients (Algo. A2b). C,D: User-dependent and sensor position-independent parameterisation by averaging parameters across body segments (Algo. A2c). UL: Upper leg, LL: Lower leg, S: Shoe.

Figure. S7 shows the 4-phase full range gait event nMAE (see Fig. 8 in main publication) between ground truth and predicted gait events of all simulated sensor positions on both body sides PRE intervention and POST intervention.

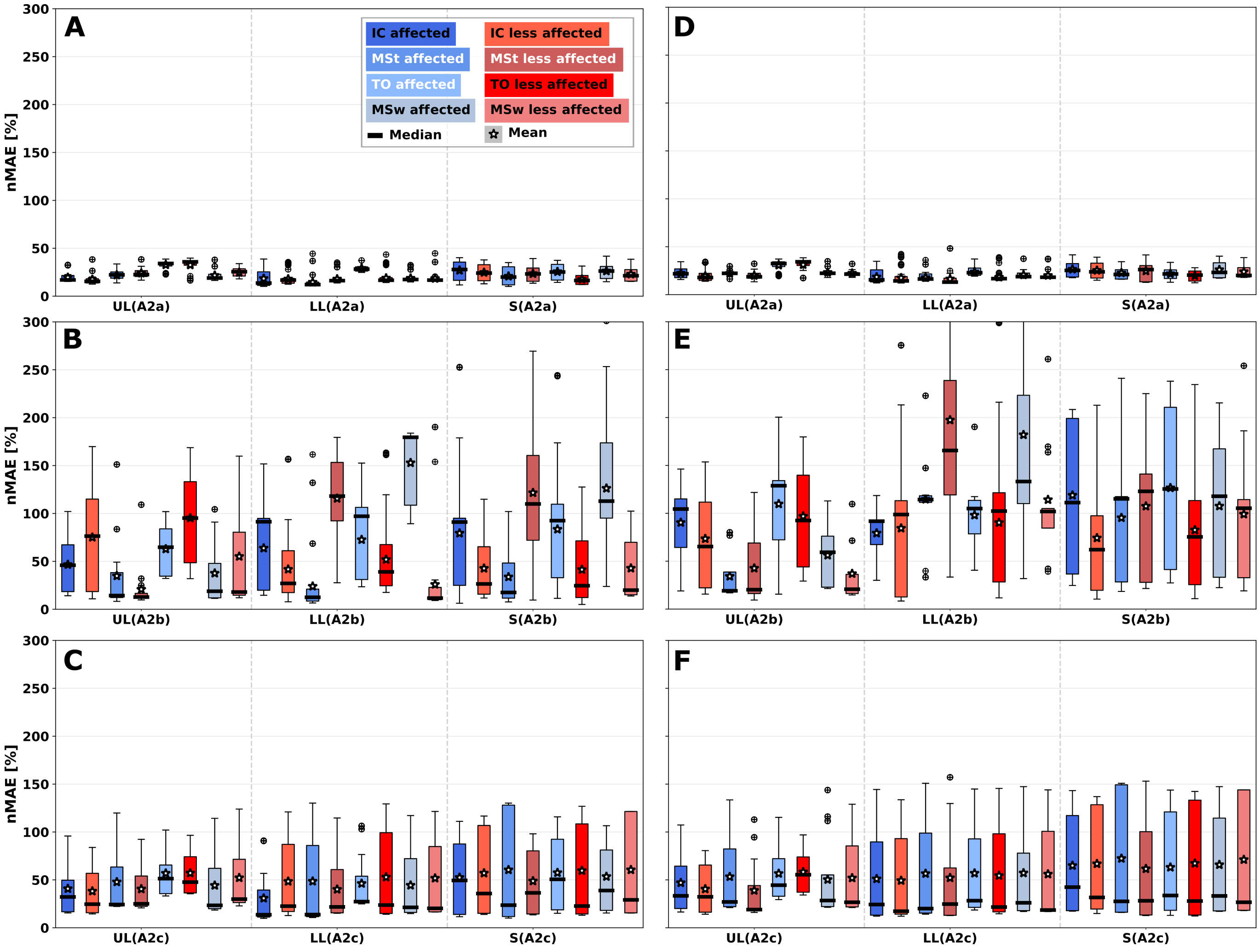

Figure S7. Full range illustration of 4-phase gait event nMAE of all simulated sensor positions summarised per body segment for all algorithms. A1 shows larger nMAE in medians for all segments. A-C: PRE intervention. D-F: POST intervention. A,D: User-dependent and sensor position-dependent parameterisation (Algo. A1, Algo A2a). B,E: User-independent parameterisation by averaging parameters across training set patients (Algo. A2b). C,D: User-dependent and sensor position-independent parameterisation by averaging parameters across body segments (Algo. A2c). UL: Upper leg, LL: Lower leg, S: Shoe.

Figure S8 show the exemplary nMAE heatmaps POST intervention on the less-affected body side for Algo. A2b and Algo. A2c for the 1-phase model.

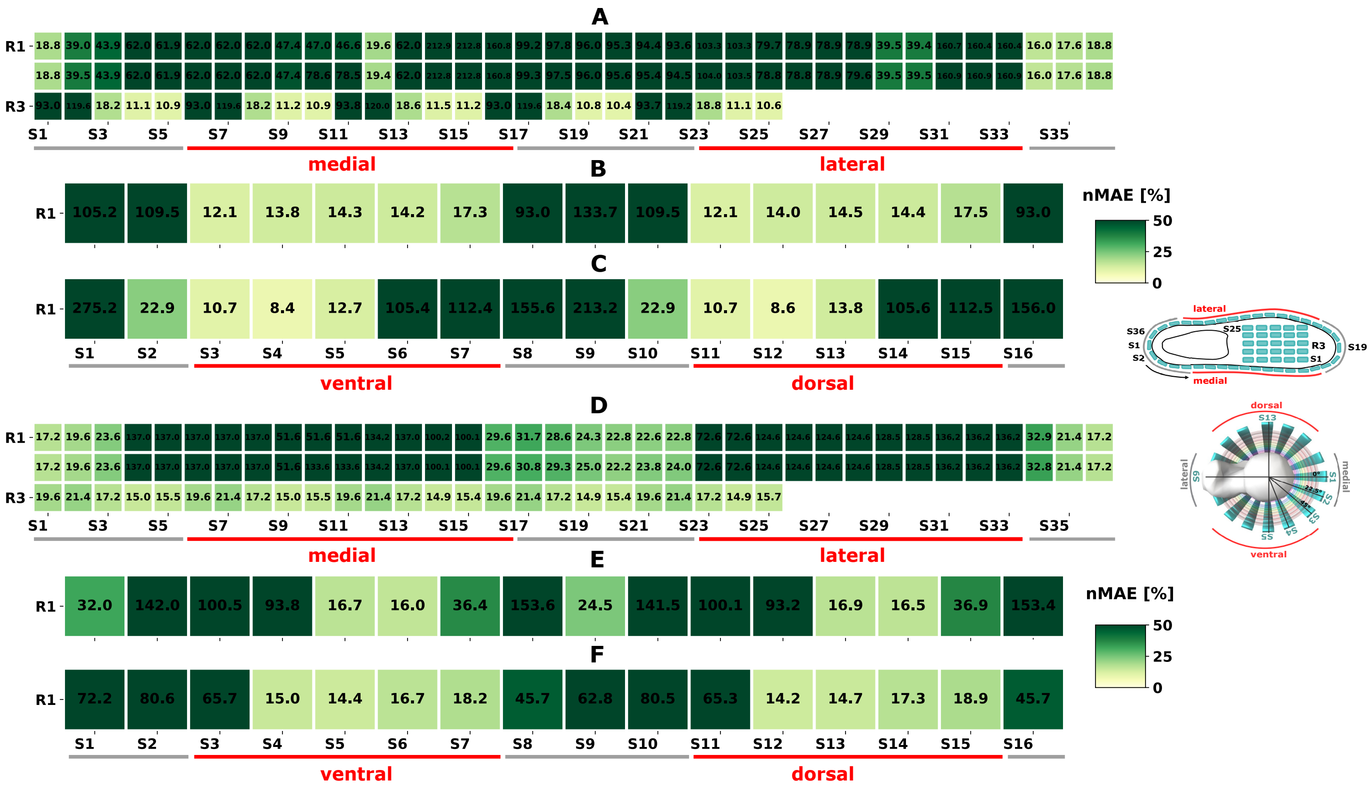

Figure S8. 1-phase model: Exemplary nMAE heatmaps POST intervention on the less-affected body side for Algo. A2b and Algo. A2c. A: All simulated shoe sensor positions forAlgo. A2b . B: Selected sensor ring at the upper leg for Algo. A2b . C: Selected sensor ring at the lower leg for Algo. A2b . D: All simulated shoe sensor positions for Algo. A2c. E: Selected sensor ring at the upper leg for Algo. A2c. F: Selected sensor ring at the lower leg for Algo. A2c. R: Ring. S: Sensor.

Figure S9 and S10 show stride duration nMAE heatmaps of all simulated sensor positions at upper and lower legs of both body sides, as well as PRE and POST intervention, for Algo. A1 and Algo. A2a.

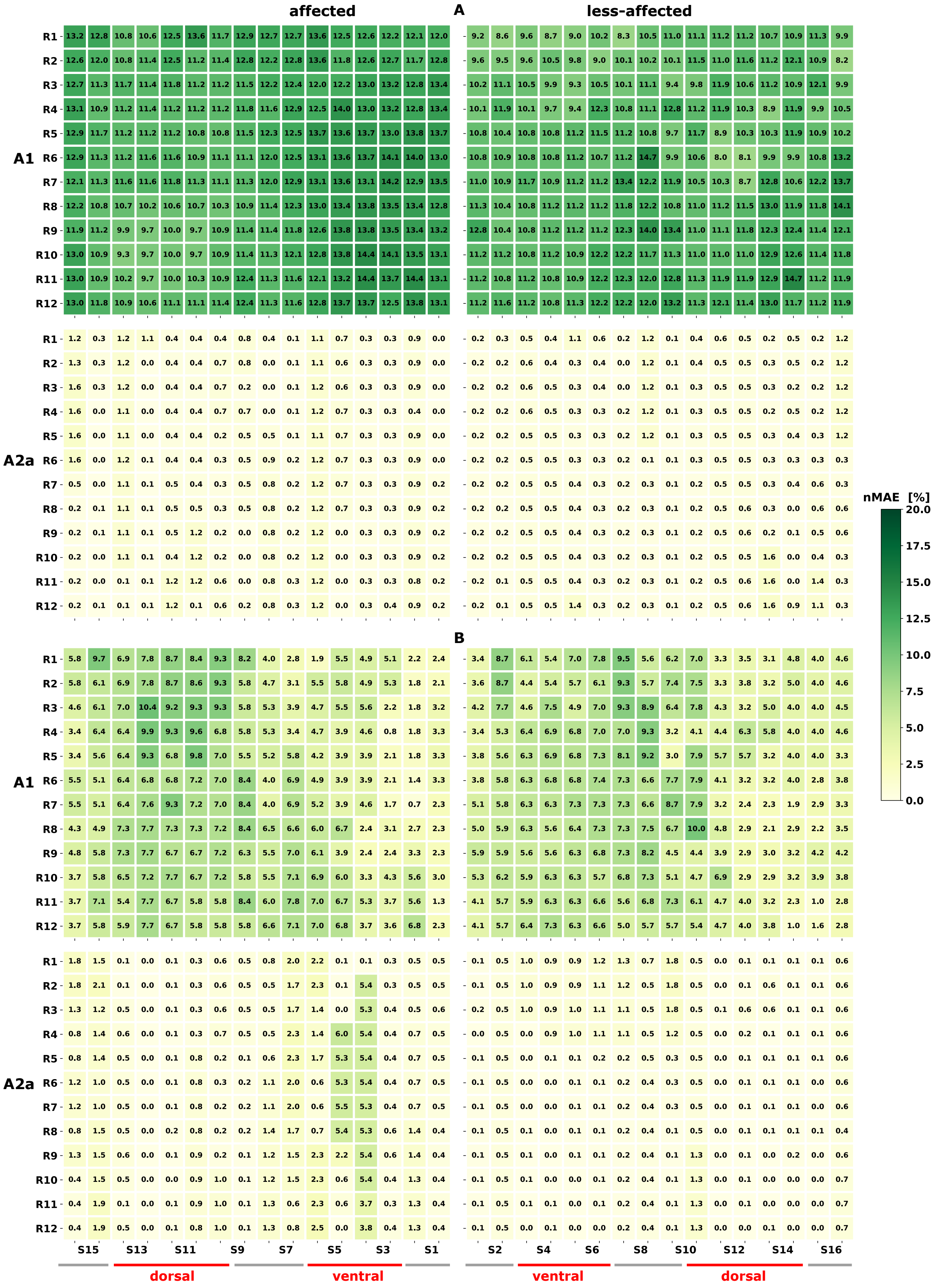

Figure S9. Stride duration nMAE heatmaps of upper leg positions at affected and less-affected body sides for Algo. A1 and Algo. A2a. A: PRE intervention. B: POST intervention. R: Ring. S: Sensor

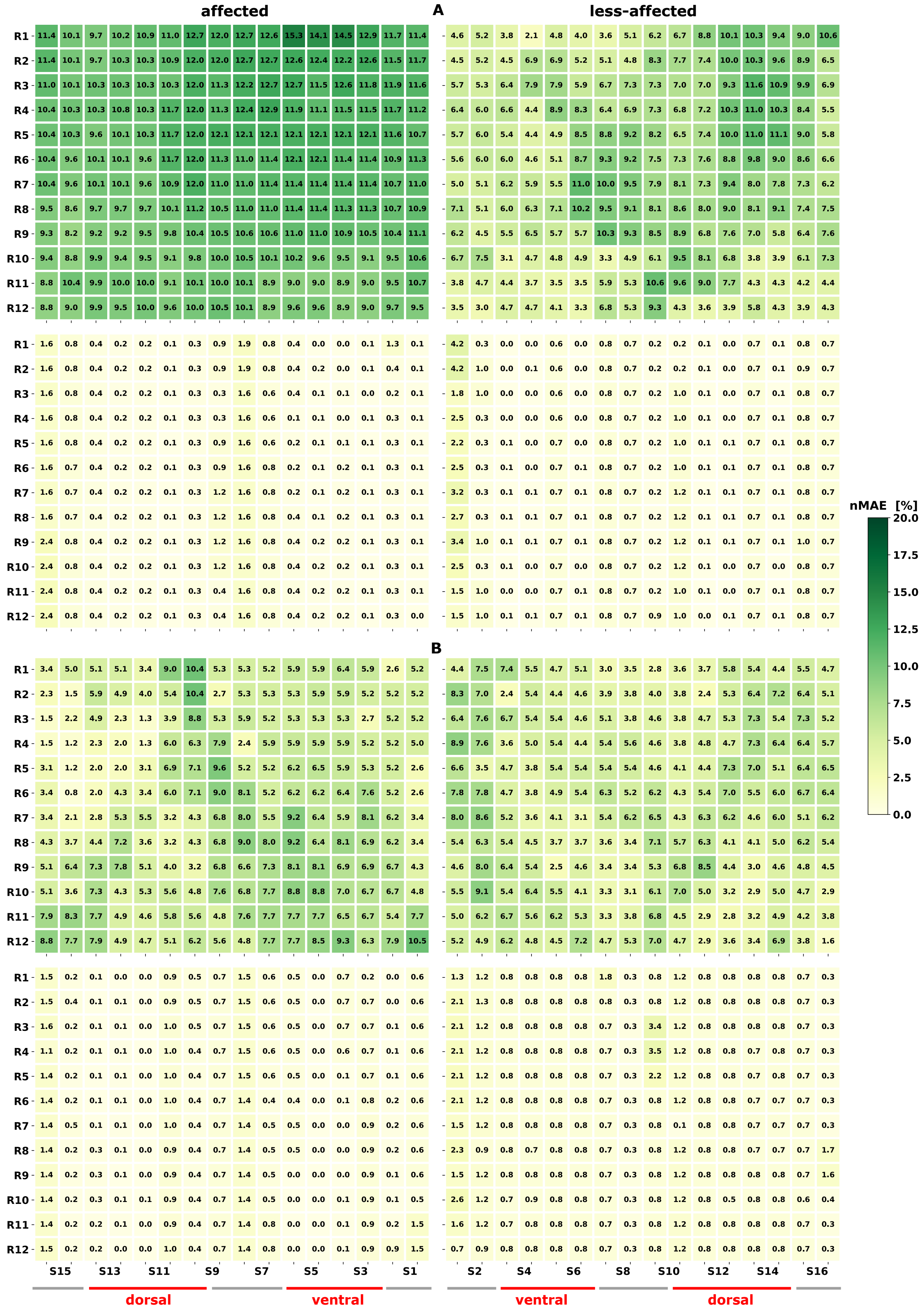

Figure S10. Stride duration nMAE heatmaps of lower leg positions at affected and less-affected body sides for Algo. A1 and Algo. A2a. A: PRE intervention. B: POST intervention. R: Ring. S: Sensor

Figure S11 shows nMAE heatmaps comprising all simulated shoe sensor positions at the affected body side.

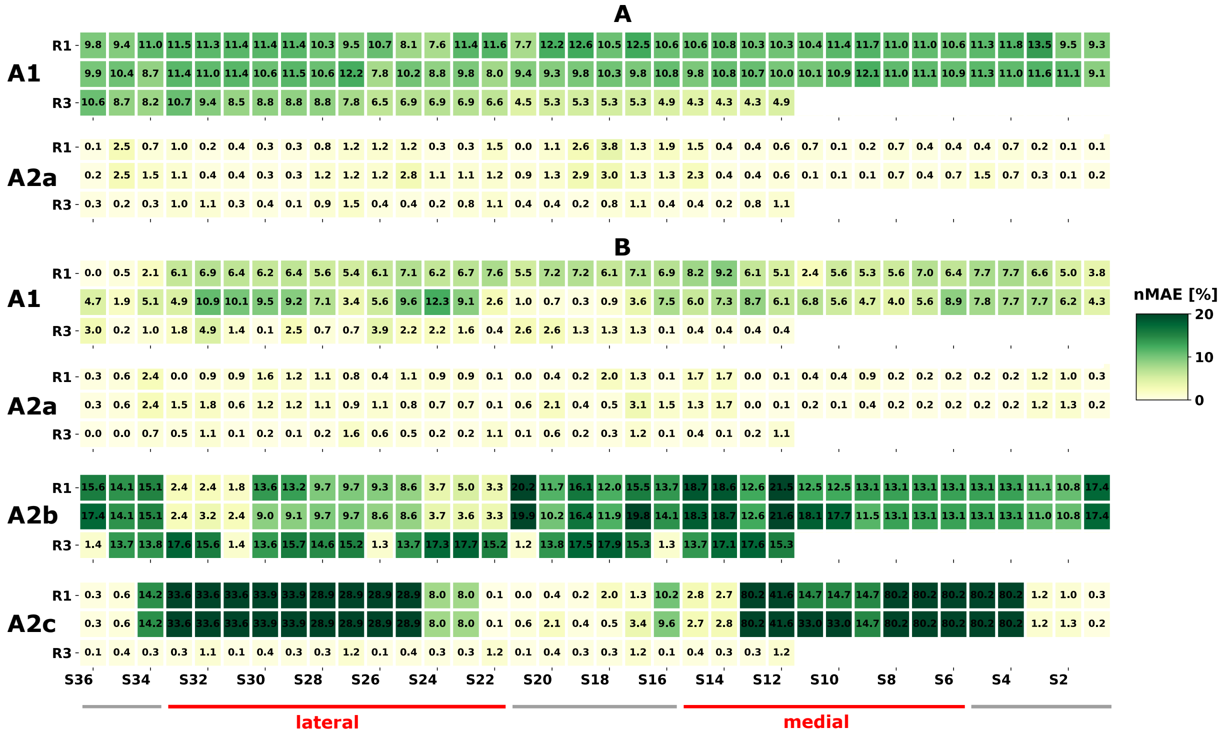

Figure S11. Stride duration nMAE heatmaps of shoe positions at the affected body side for Algo. A1 and three variants of Algo. A2a. A: PRE intervention. B: POST intervention. A2a: Baseline, parameter personalisation and position adaptation. A2b: Removing personalisation by averaging parameters across training set patients. A2c: Removing sensor position adaptation by averaging parameters across body segments. R: Ring. S: Sensor

Table S2 shows nMAEs median and interquartile range (IQR) for all body segments, at PRE and POST intervention of Algo. A1 and Algo. A2 variants for gait event detection for the 1-phase model.

Table S2. IC detection in 1-phase gait model. Quantification of nMAE median and IQR for all body segments PRE and POST intervention of Algo. A1 and Algo. A2 variants. Aff.: Affected body side. LessAff.: Less-affected body side.

			UL				LL				S			
Inter-vention	Gait Event	Algo.	Median [%] Aff.	IQR [%] Aff.	Median [%] LessAff.	IQR [%] LessAff.	Median [%] Aff.	IQR [%] Aff.	Median [%] LessAff.	IQR [%] LessAff.	Median [%] Aff.	IQR [%] Aff.	Median [%] LessAff.	IQR [%] LessAff.
PRE	IC	A1	31.8	3.6	33.9	4.2	25	1.8	35.1	2	27.9	6.9	31.8	4.3
		A2a	18.9	6.2	18.1	4.1	15.4	15.7	19.8	6	30.7	24.5	28.9	22.7
		A2b	53.9	56.9	88.8	111.6	105.9	85.9	32	50.3	106.5	84.3	32.7	57.1
		A2c	32.8	30.2	26.5	38.5	15.4	26.7	24.4	77.4	83	105.5	52.4	104.7
POST	IC	A1	25.2	5.1	29.7	4	24.3	1.4	29.6	3.9	28.5	9.4	27.5	1.7
		A2a	20.5	6.7	17.4	6.7	16.6	13.2	14.3	6	25.2	14.2	26.1	18
		A2b	104.9	50.5	65.2	89.4	91.6	25.3	98.8	99.8	110.7	163.4	62	80.3
		A2c	31.8	47.6	33.9	40.5	24.7	61.4	14.3	77.7	77	121.4	55.6	117.9

Figure. S12 exemplifies detection errors for two selected sensor positions.

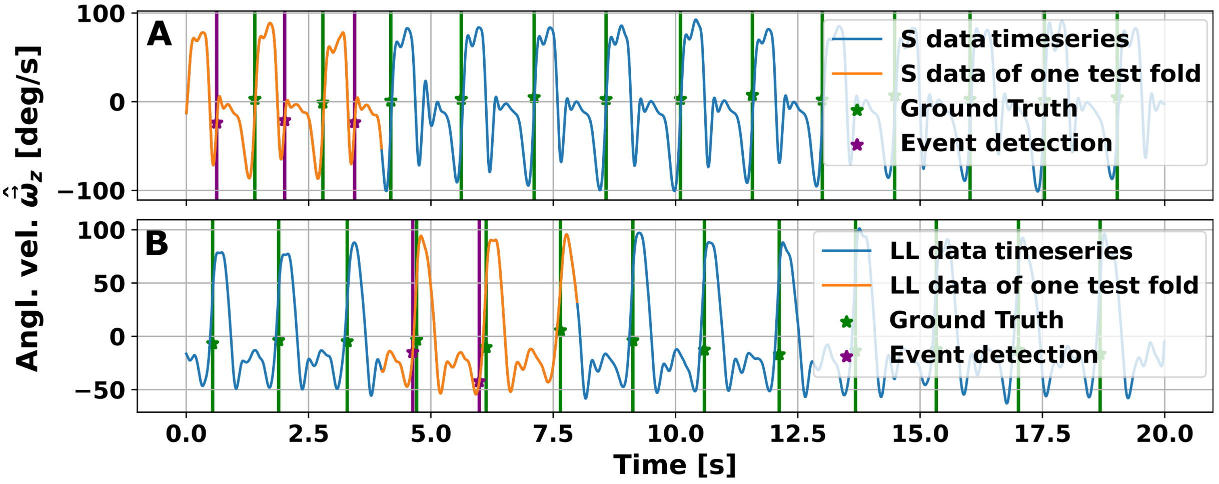

Figure S12. Example sensor timeseries illustrating 1-phase event detection errors of Algo A2c using 5-fold CV with detections for one test fold superimposed on the signal. The graph shows timing errors between IC event detection and ground truth. A: Gyroscope (Angl. vel.) timeseries of synthesis $\hat{\vec{\omega}}_z$ at a selected shoe sensor position (S). B: Gyroscope (Angl. vel.) timeseries of synthesis $\hat{\vec{\omega}}_z$ at a selected lower leg sensor position (LL).

Figure S13 and Fig. S15 show nMAE heatmaps of simulated sensor positions of both body sides, PRE and POST intervention for Algo. A2a for the 4-phase model. Due to the symmetrical sensor distribution around lower and upper legs, results for one ring of simulated sensor positions per leg segment and gait event are shown.

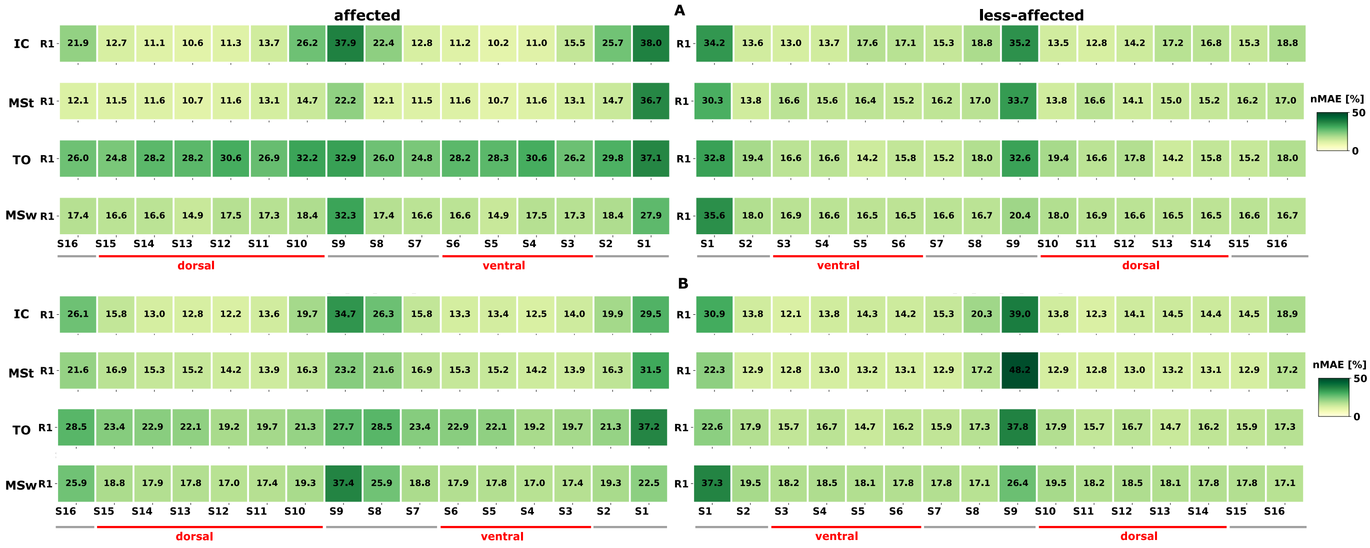

Figure S13. nMAE heatmaps of detected events with Algo. A2a for the 4-phase model, including affected and less-affected body sides. Due to the symmetrical sensor distribution around the upper leg, results for one ring of simulated sensor positions are shown. A: PRE intervention. B: POST intervention. R: Ring. S: Sensor

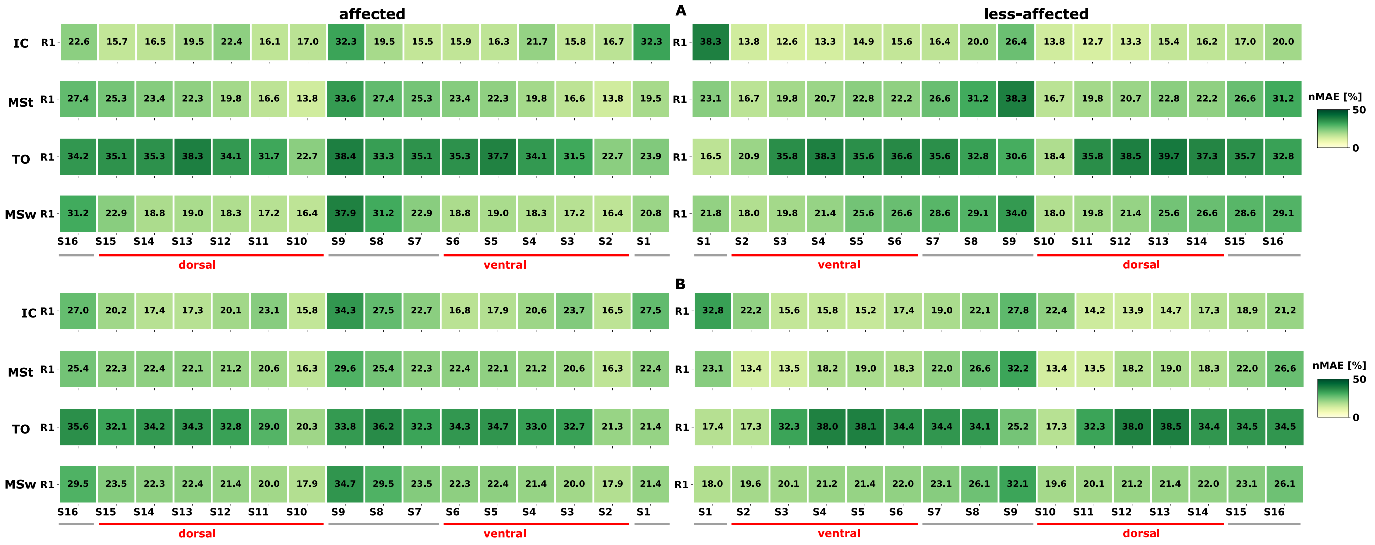

Figure S14. nMAE heatmaps of detected events with Algo. A2a for the 4-phase model, including affected and less-affected body sides. Due to the symmetrical sensor distribution around the lower leg,results for one ring of simulated sensor positions are shown. A: PRE intervention. B: POST intervention. R: Ring. S: Sensor

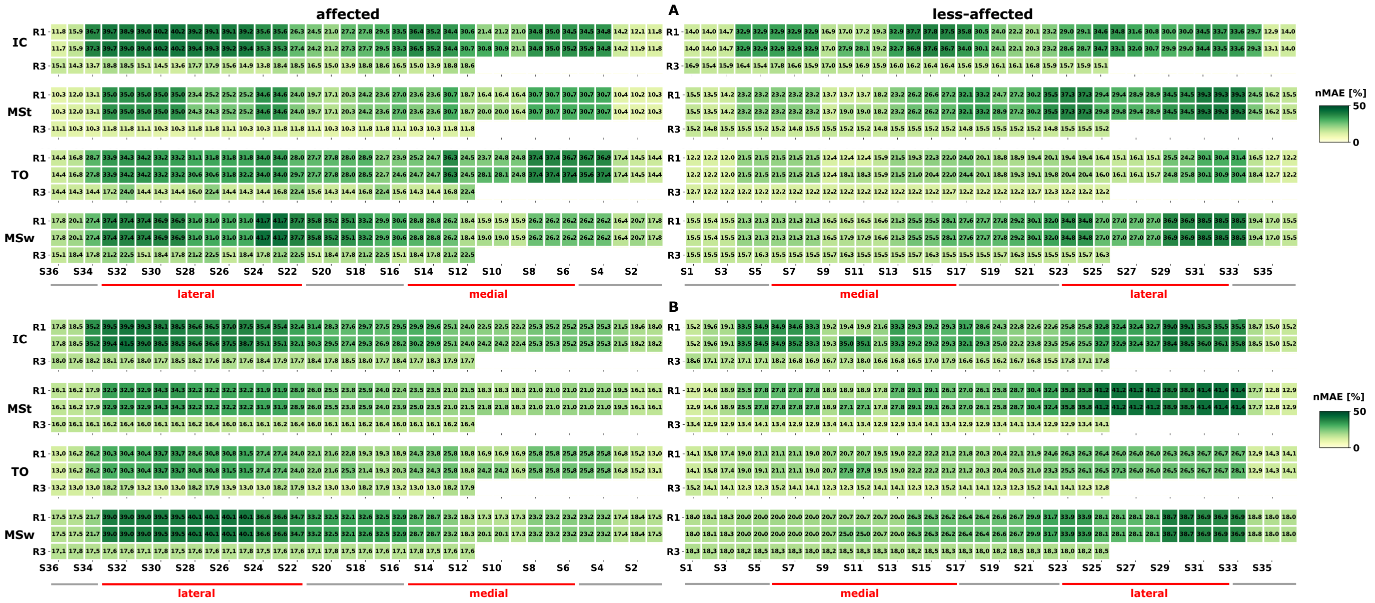

Figure S15. nMAE heatmaps of detected events with Algo. A2a for the 4-phase model, including affected and less-affected body sides at the shoe. A: PRE intervention. B: POST intervention. R: Ring. S: Sensor

Table S3 shows median and IQR for all body segments, PRE and POST intervention of Algo. A1 and Algo. A2 variants for the 2-phase model.

Table S3. 2-phase gait event detection. nMAE median and IQR for all body segments, PRE and POST intervention of Algo. A2 variants. Aff.: Affected body side. LessAff.: Less-affected body side.

			UL				LL				S			
Inter-vention	Gait Event	Algo.	Median [%] Aff.	IQR [%] Aff.	Median [%] LessAff.	IQR [%] LessAff.	Median [%] Aff.	IQR [%] Aff.	Median [%] LessAff.	IQR [%] LessAff.	Median [%] Aff.	IQR [%] Aff.	Median [%] LessAff.	IQR [%] LessAff.
PRE	IC	A2a	18.9	6.2	18.1	4.1	15.4	15.7	19.8	6	30.7	24.5	28.9	22.7
		A2b	53.9	56.9	88.8	111.6	105.9	85.9	32	50.3	106.5	84.3	32.7	57.1
		A2c	32.8	30.2	26.5	38.5	15.4	26.7	24.4	77.4	83	105.5	52.4	104.7
	TO	A2a	39.4	5.8	38.7	5.8	29	1.4	19.7	5.8	27.7	14.6	21.8	9.1
		A2b	56	29	80.8	81.2	64.6	40	33.8	20.9	61.8	82.7	28.8	42
		A2c	49.1	28.7	50.1	31.6	32	23.1	22.4	85.6	83.4	106.1	39.7	110.2
POST	IC	A2a	20.5	6.7	17.4	6.7	16.6	13.2	14.3	6	25.2	14.2	26.1	18
		A2b	104.9	50.5	65.2	89.4	91.6	25.3	98.8	99.8	110.7	163.4	62	80.3
		A2c	31.8	47.6	33.9	40.5	24.7	61.4	14.3	77.7	77	121.4	55.6	117.9
	TO	A2a	31.7	4.1	31.9	4.6	22.4	6.8	16.8	4.1	18.3	10.4	20.7	11.1
		A2b	88.4	54.3	68.1	55.5	101.9	58.3	58	50.9	73.1	78.4	53	49.5
		A2c	39.6	35.4	44.9	28.6	26.5	52.4	16.8	68.7	75.4	110	49.2	117

Figure S16 shows synthesised angular velocity time series signals for different sensor positions at the lower leg (see Fig. S16A) and shoe (see Fig. S16B).

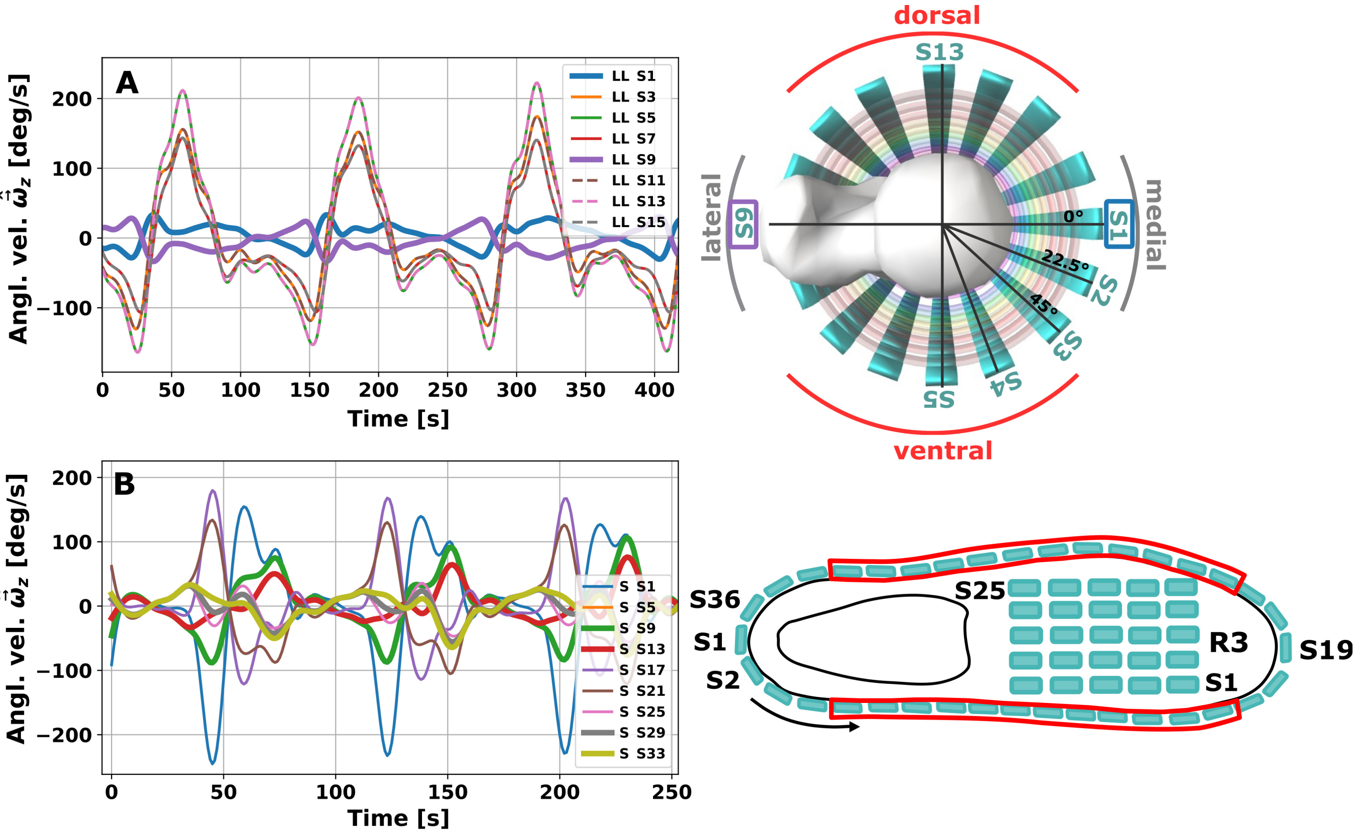

Figure S16. Gyroscope (Angl. vel.) timeseries of synthesis $\hat{\vec{\omega}}_z$ at the lower leg (A) and shoe (B). For sensor positions medial and lateral at the lower leg, the signal does not show relevant patterns, needed to detect events with the chosen algorithms. Similarly, lateral and medial shoe sides lack appropriate signal patterns required by the selected algorithms.

Table S4 shows nMAEs median and IQR for all body segments, PRE and POST intervention of Algo. A1 and Algo. A2 variants for the 4-phase model.

Table S4. 4-phase gait event detection. Quantification of nMAE median and IQR for all body segments, PRE and POST intervention of Algo. A2 variants. Aff.: Affected body side. LessAff.: Less-affected body side.

			UL				LL				S			
**Inter-vention**	**Gait Event**	**Algo.**	**Median [%] Aff.**	**IQR [%] Aff.**	**Median [%] LessAff.**	**IQR [%] LessAff.**	**Median [%] Aff.**	**IQR [%] Aff.**	**Median [%] LessAff.**	**IQR [%] LessAff.**	**Median [%] Aff.**	**IQR [%] Aff.**	**Median [%] LessAff.**	**IQR [%] LessAff.**
**PRE**	**IC**	**A2a**	19.8	6.1	17.7	4.3	15.7	17.3	19.7	6.1	32.4	25.6	28.1	20.3
		**A2b**	53.6	56.5	88.4	111.7	105.9	85.5	31.2	50.5	105.2	82.6	30.6	57.1
		**A2c**	37.3	38.1	28.5	47.6	15.8	32.7	26.1	81.1	57.3	85.2	41.4	106.1
	**MSt**	**A2a**	25.8	7.6	26	7.1	14	3.5	19	1.6	23.1	21.8	26.9	16.1
		**A2b**	16.5	30.2	14.7	5.8	14.5	14.5	136.7	70.6	20.3	42.5	127.2	102.4
		**A2c**	28.2	47.2	29	36.7	16	86.1	25.2	52.3	27.4	134.7	42.2	76.3
	**TO**	**A2a**	39.9	4.3	41.4	5.2	32.7	6.2	19.2	4.1	30	19.8	20.6	10.8
		**A2b**	75.2	57.9	110.2	98	112.5	87.4	45	51.2	107.9	88.9	28.5	68.4
		**A2c**	59.5	34.7	55.1	43.7	31.7	31.1	27.5	97.6	58.6	85.5	26.3	108.8
	**MSw**	**A2a**	21.9	5.6	29.6	8.8	20.2	2.1	19.4	1.6	30.4	14.6	24.7	13.9
		**A2b**	21.8	41.7	20.8	76.2	208	82.9	13.5	14.9	130.8	91.1	23.1	63.4
		**A2c**	27.1	48.8	34.6	52.3	24.7	65	23.5	78.9	45.1	73.1	33.7	122.5
**POST**	**IC**	**A2a**	21.8	9.1	17.8	7.4	15.5	12.7	14.3	5.1	25.3	14	23.8	15.5
		**A2b**	104.5	50.7	65.3	89.5	91.8	25	98.7	100.5	111.2	162.4	62	77.8
		**A2c**	33.4	44.2	32.4	49.2	24.3	76.5	17.3	78.9	42.5	99.2	31.7	108.9
	**MSt**	**A2a**	22.2	1.4	18.7	5.2	16.3	6.5	13.1	4.2	21	9.8	26.1	17
		**A2b**	19.1	20.3	20.3	52.8	114.3	6.9	165.6	119.4	115.1	89.1	122.9	113
		**A2c**	27	60.3	19	25.8	20.1	83.5	24.8	49.4	27.6	133	28.2	87
	**TO**	**A2a**	32.4	5	34.3	5.1	22.9	7.6	16.7	1.9	21.6	9	20.4	10.5
		**A2b**	128.8	61.9	92.5	95.7	105	35	102.3	93.2	125.4	169.3	75.4	87.8
		**A2c**	44.5	39.2	55.4	36.8	28.3	71.5	21.7	81.5	33.7	102.7	27.9	119.9
	**MSw**	**A2a**	22.3	2.5	21.4	3	18.8	4.7	18.2	1.7	23.2	15.6	20	9.9
		**A2b**	59.3	53.3	20.7	20	133.2	113.1	101.9	20.5	117.8	134	105.3	81.5
		**A2c**	28.5	33	26.7	63.6	26.1	60.1	18.5	82.9	33.3	96.9	26.7	125.7

For direct comparison with other investigations, stride duration estimates were additionally analysed by root mean squared error (RMSE) across all U_s strides per test fold. Normalised RMSE (nRMSE) was obtained in percent by normalisation with the average reference stride duration $\overline{F}$ per body side, intervention condition, and patient according to:

$$RMSE = \sqrt{\frac{1}{U_s}\sum_{i=1}^{U_s}((t_i - t_{i-1}) - (\hat{t}_i - \hat{t}_{i-1}))^2} \; ; \; nRMSE = \frac{RMSE}{\overline{F}} \cdot 100\%. \qquad \text{(S2)}$$

Average reference stride duration $\overline{F}$ was determined according to GRF reference signal (see Sec. 5.2). Table S5 shows nRMSEs and nMAEs of best and worst performing sensors per body segment for stride duration estimation, PRE and POST intervention.

Table S5. Stride duration estimation. Comparison of relative errors (nRMSE and nMAE) of best and worst performing sensor per body segment, PRE and POST intervention of Algo. A1 and Algo. A2 variants. Aff.: Affected body side. LessAff.: Less-affected body side.

			UL Best		UL Worst		LL Best		LL Worst		S Best		S Worst	
**Inter-vention**	**Algo.**	**Body side**	**nRMSE [%]**	**nMAE [%]**	**nRMSE [%]**	**nMAE [%]**	**nRMSE [%]**	**nMAE [%]**	**nRMSE [%]**	**nMAE [%]**	**nRMSE [%]**	**nMAE [%]**	**nRMSE [%]**	**nMAE [%]**
**PRE**	**A1**	**Aff.**	32.4	16.0	36.1	21.4	11.7	4.0	25.6	13.6	10.3	4.2	34.0	17.9
		**LessAff.**	17.0	7.0	30.0	16.2	1.5	0.4	26.3	12.9	7.4	2.0	31.8	18.0
	**A2a**	**Aff.**	1.1	0.0	8.0	3.7	0.5	0.0	13.2	5.6	0.3	0.0	18.1	7.7
		**LessAff.**	0.6	0.0	5.5	2.7	0.2	0.3	12.9	1.7	0.4	0.0	11.5	4.5
	**A2b**	**Aff.**	20.3	5.3	104.4	56.0	21.2	3.1	83.7	37.8	17.8	0.1	106.2	42.4
		**LessAff.**	1.1	0.1	70.3	33.8	0.9	0.3	83.7	41.7	1.6	0.0	58.7	22.5
	**A2c**	**Aff.**	1.1	0.1	83.1	69.3	0.5	0.0	92.4	58.9	0.6	0.3	95.8	48.6
		**LessAff.**	0.8	0.0	97.4	58.0	0.3	0.0	108.2	100.0	0.9	0.0	90.9	83.6
**POST**	**A1**	**Aff.**	10.6	0.2	26.8	12.2	0.5	0.0	22.4	8.7	0.7	0.0	26.5	10.0
		**LessAff.**	0.2	0.1	22.9	9.2	0.4	0.0	15.1	6.1	0.3	0.0	25.2	277.3
	**A2a**	**Aff.**	0.2	0.0	13.9	4.1	0.2	0.0	6.6	2.0	0.2	0.0	13.5	4.4
		**LessAff.**	0.1	0.0	5.7	2.2	1.8	0.3	14.0	5.9	0.2	0.0	10.0	347.6
	**A2b**	**Aff.**	16.9	0.2	74.0	22.3	27.2	0.2	83.6	20.0	25.4	1.5	79.8	27.0
		**LessAff.**	21.1	2.8	102.2	27.6	21.0	4.0	79.6	42.2	20.5	1.1	83.3	33.1
	**A2c**	**Aff.**	0.3	0.0	107.3	47.8	0.2	0.0	103.9	100.0	0.2	0.1	103.9	100.0
		**LessAff.**	0.1	0.0	84.2	58.2	1.8	0.0	117.0	61.3	0.3	0.0	103.9	100.0
